# A Cascaded Quantized Spiking Neural Network for Real-Time ECG Arrhythmia Detection on Edge Hardware

**DOI:** 10.3390/s26123723

**Published:** 2026-06-11

**Authors:** Olamilekan Banjo, Behnaz Ghoraani

**Affiliations:** Department of Electrical Engineering and Computer Science, Florida Atlantic University, Boca Raton, FL 33431, USA; obanjo2023@fau.edu

**Keywords:** ECG arrhythmia classification, quantized spiking neural networks, FPGA acceleration, edge computing, wearable health monitoring

## Abstract

Wearable ECG monitors enable continuous cardiac surveillance, but most still rely on cloud-based analysis with limited on-device support for multi-class arrhythmia detection. Spiking neural networks (SNNs) are promising for low-power edge inference, yet it remains unclear how class-imbalance loss design interacts with RR-interval features in directly trained quantized SNNs, and FPGA validation in this setting is largely unexplored. We propose a quantized convolutional spiking neural network (QCSNN) for real-time arrhythmia detection on resource-constrained hardware. The model uses a dual-head architecture that jointly trains binary and four-class classifiers, subsequently reorganized into a cascaded pipeline that routes only abnormal beats to the second stage. At inference, beats classified as Normal exit at Stage 1; only beats classified as Abnormal are routed to the four-class head, so the bulk of the inference cost is absorbed by Stage 1. We evaluate two loss functions, Cross-Entropy and Focal Loss, under four RR-feature routing strategies. Without RR features, Focal Loss improves macro F1 by 2.3–2.5% over Cross-Entropy (mean Δ = +0.013 in Stage-2 macro F1; Wilcoxon two-sided *p* = 0.031). With RR features, this advantage largely disappears (Wilcoxon two-sided *p* ≥ 0.219 at all RR routings); meanwhile, RR features at the strongest routing improve Stage-2 macro F1 by +0.028 to +0.034 depending on loss function—a gain that exceeds the entire Focal-Loss-over-Cross-Entropy advantage, suggesting that RR features provide discriminative information that compensates for class imbalance at the input level. Based on clinically prioritized sensitivity, the CE:RR→Both configuration was deployed on a PYNQ-Z2 FPGA, achieving 99.02% cascaded accuracy, 11.54 ms per-beat latency, and 0.33 W accelerator power—a 31.66× power reduction and 4.01× energy reduction versus GPU inference, within 1% macro F1. These results demonstrate quantized SNNs as a practical solution for real-time edge arrhythmia monitoring that operates independently of cloud connectivity—removing the network-dependent latency, connectivity-dropout failure modes, and continuous-transmission energy burden that constrain current wearable monitors and, to our knowledge, represent one of the first systematic studies of loss-function/RR-feature interactions in directly trained SNN arrhythmia classification and one of the first FPGA deployments of a fully quantized, directly trained SNN for multi-class ECG arrhythmia detection. All code generated and used in this study has been made publicly available.

## 1. Introduction

Cardiovascular diseases (CVDs) remain the leading cause of death globally, claiming an estimated 17.9 million lives annually. Among these, cardiac arrhythmias—disorders of the heart’s electrical conduction system that produce irregular rhythms—are particularly dangerous, as they significantly elevate the risk of stroke, heart failure, and sudden cardiac death. Early detection and treatment can substantially reduce mortality and prevent acute cardiac events, yet timely diagnosis remains challenging due to the transient and often unpredictable nature of arrhythmic episodes.

Traditional arrhythmia detection uses clinical electrocardiograms (ECGs), providing only brief, episodic heart snapshots which can miss critical changes in a patient’s arrhythmia condition that can occur in between check-ups or outside visiting hours, without immediate notice. Wearable health devices such as smartwatches and smart stethoscopes address these shortcomings by enabling clinicians to view physiological data in real time and intervene when abnormalities arise [1]. Consumer- and medical-grade wearables continuously track physiology and activity during daily life [1], extending monitoring beyond the hospital into the patient’s natural environment. By capturing day-to-day metrics between visits, these tools provide a more complete picture of cardiac health status.

Despite expanding access to arrhythmia monitoring, current wearable ECG platforms—such as the Apple Watch, AliveCor KardiaMobile, and QardioCore—still largely follow a cloud-centric pipeline: the device records a single-lead ECG (or PPG) and transmits derived metrics to a smartphone or cloud server for interpretation. On-device analytics remain limited to basic measures such as instantaneous heart rate and, at best, coarse detection of atrial fibrillation, rather than reliable multi-class identification of subtler arrhythmias [2,3]. This fully centralized design introduces practical drawbacks: continuous data acquisition and transmission accelerates battery depletion, while network dependence adds latency and connectivity dropouts—making response time unreliable and poorly suited for time-critical arrhythmia detection. Deploying more advanced AI directly on-device could address these limitations but is itself constrained by the tight battery, memory, and computational budgets of wearable hardware. A successful on-device classifier therefore does more than offload computation: it decouples the wearable from the cloud entirely, eliminating network-dependent latency, preserving function during connectivity loss, and removing the continuous transmission burden that drives battery drain.

To address these challenges, there is growing interest in developing edge AI solutions—intelligent algorithms that can operate directly on-device. Among emerging approaches, spiking neural networks (SNNs) stand out as a particularly compelling candidate for on-device ECG analysis. As third-generation neural networks, SNNs encode and transmit information as discrete spikes rather than continuous real-valued activations, enabling event-driven computation that is inherently sparse and energy-efficient [4]. Critically, the leaky integrate-and-fire (LIF) neuron at the core of most SNN architectures maintains a decaying membrane potential that accumulates incoming signals over time—a temporal integration mechanism that naturally aligns with the sequential, time-varying nature of ECG waveforms [5]. This intrinsic capacity to capture temporal dynamics means that SNNs can exploit the morphological evolution within a heartbeat (P-wave, QRS complex, T-wave transitions) without the explicit recurrence or attention mechanisms that conventional DNNs require [5]. Furthermore, because spiking neurons only compute when spikes occur, inference on predominantly normal ECG beats—where activation is sparse—incurs minimal computational cost, making SNNs well suited for always-on monitoring on battery-constrained wearable hardware [6]. While SNN-based ECG classification is a maturing area and effective training remains challenging, recent advances in surrogate gradient methods have made direct training of deeper SNN architectures increasingly practical [4].

A key design choice in training any neural network classifier—including SNNs—is the loss function. Most SNN-based ECG classification studies default to cross-entropy (CE) loss, which optimizes overall classification accuracy but can neglect minority classes when training data is heavily imbalanced [7]. The MIT-BIH Arrhythmia Database exemplifies this problem: Normal (N) and Ventricular Ectopic Beats (VEBs) dominate the dataset, while Supraventricular Ectopic Beats (SVEBs) and Fusion (F) beats are severely underrepresented, causing CE-trained models to achieve high overall accuracy while performing poorly on clinically important rare arrhythmias. Focal Loss (FL), which down-weights easily classified samples to refocus training on difficult minority cases, has been applied in ECG classification specifically to address this imbalance [7,8]. Separately, combining morphological (waveform) features with RR-interval features is a well-established strategy in conventional deep learning models for ECG classification [9,10]. RR intervals capture inter-beat timing dynamics—heart rate variability, prematurity, and compensatory pauses—that the local morphological window alone may miss, and are clinically critical for distinguishing SVEBs and VEBs from normal beats [9]. Notably, both FL and RR-interval features target the same underlying challenge—improving detection of underrepresented arrhythmia classes—but from fundamentally different angles: FL reshapes the optimization landscape, while RR features enrich the input representation. Although prior work has employed both strategies simultaneously [8], no study has systematically investigated how loss function selection and RR-interval feature inclusion interact—specifically, whether the addition of RR features alters the relative benefit of FL over CE. This interaction is particularly unexplored in the SNN domain, where the interplay between spike-based temporal processing, feature routing, and loss function dynamics may differ from conventional DNNs.

Meanwhile, *model quantization* has gained traction as a method to reduce model size and computation without significant loss in accuracy. This method reduces the numerical precision of model parameters and computations. For example, 32-bit floating point weights can be converted to 8-bit integers. Quantization dramatically lowers the memory footprint of the model and can speed up inference (especially on hardware that supports integer arithmetic), with only minor impact on accuracy [11]. This advantage is especially pronounced on FPGAs, whose reconfigurable fabric is built around fixed-point DSP slices and lookup tables—making integer-only quantized models a natural fit for FPGA-based deployment, where floating-point arithmetic would otherwise incur substantial area and power overhead. By using lower-precision representations for weights and activations, quantized models consume less energy and run faster on-device.

In this paper, we present a *quantized convolution spiking neural network (QCSNN)*—a dual-head lightweight classification architecture that is later converted to a two-stage classification model—designed for seamless integration into embedded digital health monitoring devices. The QCSNN combines the computational efficiency of model quantization with the temporal precision and sparsity of spiking neural networks, and is trained using quantization-aware training (QAT) and surrogate gradient descent to enable real-time, on-device arrhythmia detection. During training, we explored the effect of loss function choice and RR-interval feature routing strategies on model performance. The selected QCSNN model was accelerated on a PYNQ-Z2 FPGA platform, where the system first performs binary classification (Normal vs. Abnormal) and then applies refined multi-class classification to abnormal beats.

This work contributes to the broader field of AI-enabled wearables by advancing the feasibility of deploying clinically meaningful deep learning models on constrained hardware, thereby reducing reliance on cloud infrastructure. The key contributions of this work are as follows:

1. We propose a dual-head QCSNN training architecture in which binary (Normal vs. Abnormal) and four-class arrhythmia classification heads are jointly trained using surrogate gradient descent and quantization-aware training, then reorganized into a two-stage cascaded inference pipeline that performs coarse-to-fine classification.
2. We conduct a systematic ablation across eight training configurations—two loss functions (Cross-Entropy and Focal Loss) and four RR-interval feature routing strategies (No RR, RR→S1, RR→S2, RR→Both)—revealing that the inclusion of RR-interval features substantially reduces the performance advantage of Focal Loss over Cross-Entropy, suggesting that RR features compensate for the class-imbalance sensitivity that Focal Loss was designed to address.
3. The cascaded two-stage inference pipeline exploits the prevalence of normal beats in continuous monitoring to reduce average computational load by over 60%, as the majority of beats are resolved at the lightweight binary stage without invoking the deeper four-class classifier.
4. We deploy the selected configuration (CE:RR→Both) on a PYNQ-Z2 FPGA using integer-only quantized arithmetic and validate deployment fidelity by comparing classification metrics (precision, recall), latency, throughput, power consumption, and resource utilization against the GPU-based implementation.

Since the target deployment scenario is continuous personal cardiac monitoring on a wearable device—where the model would be calibrated to an individual user—we adopt a patient-specific (intra-patient) evaluation protocol with an 80/20 train–test split. This deployment model is consistent with established practice in patient-specific wearable ECG classification research [12,13], where a dedicated classifier is calibrated to an individual user during an initial enrolment phase using a small subset of patient-specific data; the intra-patient protocol is therefore matched to the target deployment, not a methodological shortcut around cross-patient generalization, which remains a distinct and complementary research direction (Section 4.8). This design simulates the intended use case in which the system observes a portion of a patient’s ECG history during an initial calibration phase and subsequently classifies future beats from the same individual. This protocol is consistent with prior work on patient-specific wearable ECG classifiers [12,13] and more faithfully reflects the operational conditions of a dedicated personal monitoring device than an inter-patient protocol, which evaluates generalization to entirely unseen patients—a different and complementary objective that falls outside the scope of this work.

Collectively, these results support next-generation smart health solutions that are autonomous, privacy-preserving, energy-efficient, and responsive—even in offline or resource-limited environments.

## 2. Related Works

Deep learning (DL) has become the dominant paradigm for automatic ECG arrhythmia classification, outperforming traditional machine learning methods such as support vector machines and random forests in mapping raw or minimally preprocessed signals to diagnostic categories [14,15]. Among DL architectures, convolutional neural networks (CNNs) have seen the widest adoption, owing to their effective hierarchical feature extraction and ease of integration into hybrid architectures [16]. However, as noted by Xiao et al. [16], the overwhelming focus of this body of work has been on maximizing classification accuracy, with limited attention to the computational and memory constraints that govern deployment on resource-limited edge devices—a critical gap given the growing demand for on-device inference in wearable cardiac monitors.

Spiking neural networks (SNNs), whose computational properties for ECG analysis were outlined in Section 1, have attracted growing attention as energy-efficient alternatives to conventional DNNs for arrhythmia classification. A number of SNN-based ECG classifiers have been proposed in recent years, differing primarily in how the spiking models are constructed and trained. Early efforts adopted an *ANN-to-SNN conversion* strategy in which a pretrained CNN is mapped onto an equivalent spiking architecture by replacing continuous activations with integrate-and-fire neurons. Yan et al. [17] employed this approach in a two-stage CNN/SNN pipeline for MIT-BIH classification, deliberately avoiding handcrafted features to minimize computational cost. Feng et al. [5] demonstrated a 14-layer deep SNN converted from a CNN counterpart, achieving accuracy comparable to the original ANN. Gilani et al. [18] and Ho et al. [19] similarly relied on conversion-based pipelines for ECG classification. However, the mismatch between continuous ANN activations and discrete SNN spikes introduces approximation errors that require careful threshold balancing and weight renormalization, often limiting classification performance [20]. To overcome these limitations, *direct training* of SNNs using surrogate gradient descent—which substitutes a differentiable approximation for the non-differentiable spike function during backpropagation—has emerged as the more effective paradigm, consistently outperforming conversion-based methods in accuracy and training stability [21]. The QCSNN architecture proposed in this work adopts direct training via surrogate gradients, and the mathematical formulation of the LIF neuron dynamics underlying our model is presented in Section 3.

Direct training of SNNs via surrogate gradients typically employs cross-entropy (CE) loss applied to output spike counts or membrane potentials aggregated across simulation timesteps [22]. While CE is effective for overall accuracy, it is well known to underperform on minority classes under severe class imbalance—a pervasive characteristic of clinical ECG datasets such as MIT-BIH, where Normal beats vastly outnumber arrhythmic categories. Several SNN-specific loss formulations have been proposed to address other training challenges: Deng et al. [23] introduced TET loss, which applies CE independently at each timestep to improve temporal generalizability; Guo et al. proposed IM-Loss [24] to maximize information flow through spike quantization layers, and RMP-Loss [25] to regularize membrane potential distributions for reduced quantization error. Notably, none of these address class imbalance directly. In the conventional DNN literature, focal loss (FL) [26] has become the standard remedy, down-weighting well-classified examples to refocus optimization on hard, typically minority-class samples. Yet its adoption in SNN-based classifiers remains sparse [27], and no SNN study has systematically compared CE and FL under varying input feature conditions to understand how loss function choice interacts with the information available to the model.

The RR interval, defined as the duration between consecutive R-peaks, is a clinically established inter-beat feature inversely proportional to heart rate [28]. RR intervals capture timing dynamics that the local morphological window cannot—including heart rate variability, beat prematurity, and compensatory pauses—and are particularly discriminative for SVEB and VEB classes that are otherwise morphologically subtle. In conventional deep learning, fusing RR-interval features with waveform representations has proven consistently effective. Berrahou et al. [29] combined 1D-CNN-extracted morphological features with RR-interval and entropy rate descriptors, achieving 98.73% inter-patient accuracy on MIT-BIH. Zhang et al. [30] fed normalized local and global RR intervals as separate channels alongside ECG segments into an adversarial CNN for subject-invariant heartbeat classification. Zhou et al. [31] integrated RR-interval features with multiscale convolutional blocks and a frequency attention module, reporting 95.6% inter-patient accuracy. Saadatnejad et al. [12] processed RR intervals jointly with wavelet features through dual lightweight LSTM models for wearable deployment, improving SVEB and VEB F1 scores by up to 15.5%. Across these works, the consistent finding is that RR features provide complementary discriminative power that morphology-only models lack, particularly for minority arrhythmia classes.

Despite this well-established practice in conventional DL, SNN-based ECG classifiers have largely omitted RR-interval features. Yan et al. [17] explicitly avoided handcrafted features including RR intervals in their two-stage CNN/SNN, operating on raw signals to minimize energy cost. Xing et al. [32] employed an SNN with channel-wise attention but relied solely on spike-encoded waveform morphology without RR augmentation. Similarly, Feng et al. [5] built a 14-layer deep SNN via ANN-to-SNN conversion using only raw ECG input. Liu et al. [6] designed a spike-driven SNN processor for wearable ECG classification using level-crossing sampling and spatio-temporal backpropagation, again without incorporating RR features. This omission is notable given the consistent improvements RR features provide in conventional models, particularly for the minority arrhythmia classes (SVEB, VEB) that SNN-based classifiers also struggle with. To date, no study has systematically investigated the integration of RR-interval features into directly trained SNNs for arrhythmia classification, nor examined how such features should be routed within multi-stage SNN architectures.

The preceding discussion reveals two independent strategies for improving minority-class arrhythmia detection: on the loss function side, focal loss reshapes the optimization landscape to prioritize difficult, typically underrepresented samples [26]; on the input representation side, RR-interval features supply complementary temporal context that is especially discriminative for SVEB and VEB beats [9]. In conventional DNN literature, these strategies have been combined—for instance, Wang et al. [8] employed both focal loss and RR features in a CNN-MLP hybrid—but always as a fixed design choice, without isolating or comparing their individual and joint effects. In the SNN domain, neither strategy has received adequate attention: focal loss remains rarely adopted [27], and RR features are routinely omitted. This leaves a fundamental question unaddressed: does the inclusion of RR-interval features alter the relative advantage of focal loss over cross-entropy, or do these two strategies provide redundant benefits for minority-class performance? Answering this question requires a controlled experimental framework that varies both loss function and RR feature routing simultaneously—an investigation that, to the best of our knowledge, has not been conducted in any architecture, conventional or spiking.

Parallel to advances in spiking architectures, *quantization* has become a key enabler for deploying neural networks on resource-constrained hardware. By representing weights and activations with low-precision integers instead of floating-point values, quantized models achieve significant reductions in memory footprint and computational cost [33]. When applied to SNNs, quantization compounds the efficiency gains already provided by spike-based computation: the sparse, binary nature of spikes combined with low-bitwidth weights and activations yields models that map naturally onto fixed-point arithmetic units in embedded processors and FPGA fabrics. Quantization-aware training (QAT), which simulates reduced-precision arithmetic during the training loop, has been shown to preserve classification accuracy more effectively than post-training quantization, particularly at aggressive bit widths (e.g., 4-bit or below) where rounding errors can otherwise degrade performance [11].

For deploying these quantized models, FPGAs have emerged as a compelling platform for real-time ECG classification, offering reconfigurable parallelism and low power consumption compared to general-purpose processors. Several works have demonstrated FPGA-accelerated CNN-based arrhythmia classifiers: Aruna et al. [34] deployed a quantized DCNN on a Xilinx FPGA achieving 99.67% accuracy on the PTB database; Tiwari and Muduli [35] implemented a deep residual CNN on a PYNQ-Z2 board achieving 95.6% accuracy with only 29,263 parameters; and Lu et al. [36] proposed an unstructured sparse CNN accelerator for wearable ECG devices achieving 98.99% accuracy with 70% sparsity. However, these FPGA deployments have exclusively targeted conventional CNN architectures. Despite the natural compatibility between SNNs’ integer spike-based computation and FPGA fixed-point arithmetic, FPGA deployment of quantized SNNs for ECG classification remains largely unexplored. Xing et al. [32] demonstrated an SNN with channel-wise attention on FPGA but without quantization-aware training, and Chu et al. [6] validated a spike-driven SNN processor in 40 nm CMOS technology rather than on a reconfigurable FPGA platform. To our knowledge, this work is among the first to deploy a fully quantized, directly trained SNN on an FPGA for multi-class arrhythmia classification with a systematic comparison of classification fidelity, latency, throughput, power, and resource utilization against a GPU baseline.

A complementary architectural strategy for reducing computational cost in continuous ECG monitoring is the *two-stage cascaded classifier*, where a lightweight first stage performs coarse screening (e.g., Normal vs. Abnormal) and a deeper second stage is invoked only for cases flagged as abnormal. This coarse-to-fine design has been applied broadly in medical diagnostics—including ECG classification across edge-cloud platforms [37], breast histology analysis [38], and ultrasound-based BI-RADS categorization [39]. In the context of SNN-based arrhythmia detection, Yan et al. [17] first applied this principle, proposing a two-stage pipeline with a binary CNN/SNN followed by a multi-class SNN, demonstrating significant computational savings. Inspired by this approach, in our prior work [40] we introduced the quantized convolution spiking neural network (QCSNN)—a two-stage architecture in which each stage was trained separately via quantization-aware training and surrogate gradient descent using snnTorch [41] and Brevitas [42]. That work demonstrated that the hierarchical two-stage configuration yields improved error correction and memory efficiency compared to standalone models. However, training the stages independently introduces a mismatch: the second stage never learns to compensate for the first stage’s errors during training, potentially limiting overall system performance.

To delineate the present work from our previous work [40], Table S1 in the Supplementary Materials summarizes the four substantive extensions that distinguish this work. Apart from the conceptual reuse of the two-stage cascaded inference structure (already standard in the literature [17]), no figure, table, equation, or experimental result is reused verbatim from the previous work. Concretely, this work introduces twelve figures and nineteen tables in the main paper, supplemented by two further tables (Tables S1 and S2). The eight-configuration loss-function/RR-feature factorial study, the FPGA deployment-and-validation analysis with GPU baseline comparison, the exploratory inter-patient ablation, and the cross-fold statistical-significance methodology are all new content not present in the conference version.

The present work addresses the gaps identified above through a unified framework, detailed in the sections that follow. The remainder of this paper is organized as follows: Section 3 describes the proposed architecture, training methodology and experimental setup, Section 4 presents and discusses the results, and Section 5 concludes the paper.

## 3. Methods

**Figure 1:**
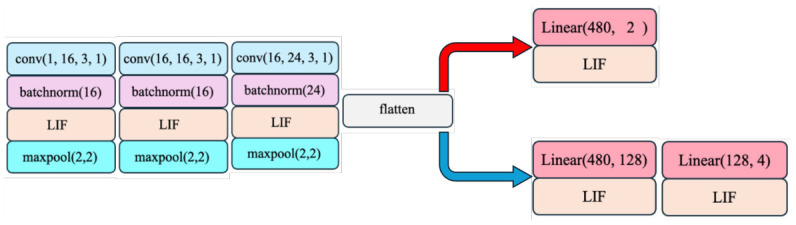
Dual-Head QCSNN structure: main body comprises the first 3 convolution blocks and the flatten layer which then branches into the binary head and the multi-class head. The heads are made of linear blocks. Each convolution block has conv → batchnorm → LIF → maxpool, while each linear block contains Linear → LIF.

**Figure 2:**
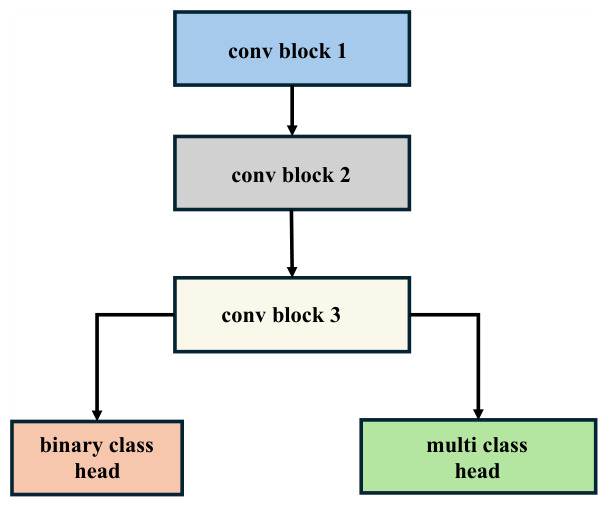
Dual-head QCSNN training architecture. The shared convolutional backbone feeds into two jointly optimized classification heads: a binary head (Normal vs. Abnormal) and a four-class head (N, SVEB, VEB, F).

**Figure 3:**
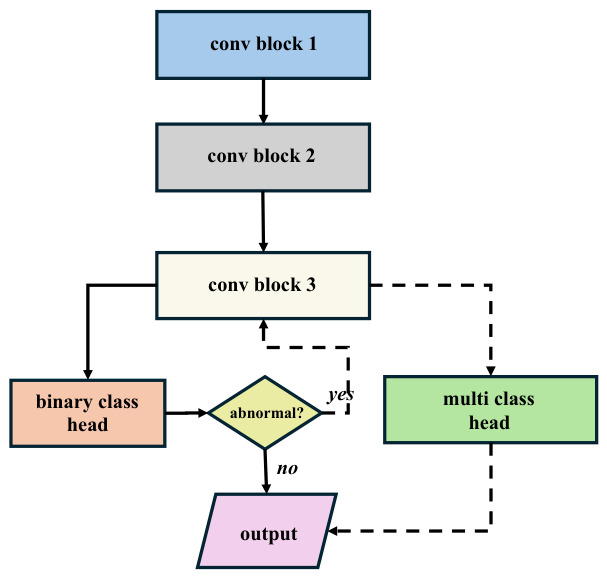
Two-stage cascaded inference architecture. The binary head (Stage-1) acts as a gate: samples predicted Normal exit immediately, while samples predicted Abnormal are routed to the multi-class head (Stage-2) for fine-grained classification.

The cascaded design philosophy is straightforward: rather than solving the multi-class arrhythmia problem in a single pass, the system first determines whether a beat is abnormal at all, and only then classifies the type of arrhythmia. This staged approach matches the prevalence structure of continuous monitoring, where most beats are normal and require no further analysis. In the MIT-BIH evaluation set, approximately 89% of beats are Normal, so in practice the cascade resolves the majority of inferences at Stage 1 without invoking the four-class head. We developed a dual-head QCSNN architecture (Figure 1) in which binary (Normal vs. Abnormal) and four-class (N, SVEB, VEB, Fusion) classification heads are jointly trained on a shared convolutional backbone, then converted to a two-stage cascaded inference pipeline for deployment on hardware-constrained platforms. During training (Figure 2), both heads are optimized simultaneously via surrogate gradient descent and quantization-aware training, enabling the shared backbone to learn feature representations beneficial for both tasks. For inference, the trained dual-head model is reorganized into a cascaded configuration (Figure 3): the binary head serves as Stage-1, classifying each beat as Normal or Abnormal; beats classified as Normal exit immediately, while beats classified as Abnormal are routed to the four-class head (Stage-2) for refined classification into N, SVEB, VEB, or Fusion—retaining a Normal class to allow self-correction of Stage-1 misclassifications. This design simultaneously addresses memory, computational, and energy constraints by limiting full-pipeline execution to abnormal samples only.

### 3.1. Dataset and Preprocessing Pipeline

#### 3.1.1. ECG Dataset and Arrhythmia Class Definition

This study employed the MIT-BIH Arrhythmia Database [43], a well-established public dataset widely used for benchmarking arrhythmia detection algorithms. This publicly available database comprises 48 ambulatory ECG recordings, each approximately 30 minutes in duration, collected from 47 individual patients. The recordings were obtained using portable Holter monitoring devices during normal daily activities, with each heartbeat subsequently labeled by expert cardiologists following the AAMI (Association for the Advancement of Medical Instrumentation) classification guidelines. The modified limb lead II (MLII) signal was chosen for analysis in this work, owing to its reliable R-peak visibility and widespread use in clinical arrhythmia assessment.

Following the AAMI standard, the classification scheme adopted in this study targets four heartbeat categories: Normal (N), representing regular sinus rhythm; Supraventricular Ectopic Beat (SVEB), arising from premature atrial or junctional activation; Ventricular Ectopic Beat (VEB), resulting from premature ventricular depolarization; and Fusion Beat (F), produced by the coincidence of supraventricular and ventricular impulses. SVEB and VEB are of particular clinical significance as potential precursors to more serious arrhythmias, while Fusion beats pose classification challenges due to their ambiguous hybrid morphology.

#### 3.1.2. Beat Segmentation and Signal Normalization

To prepare the data for input into the spiking neural network, each annotated heartbeat was segmented into a fixed-length window comprising 180 sample points centered on the R-peak. This window size was chosen to encompass the full P-QRS-T complex, capturing both pre- and post-ventricular activity, which is essential for reliable morphological classification. All ECG segments were then normalized to zero mean and unit variance, ensuring consistency in amplitude and baseline across subjects and sessions.

Baseline wandering and low-frequency drift—common artifacts in ambulatory ECG signals—were removed using a high-pass filtering step. This preprocessing phase improved the signal-to-noise ratio and facilitated robust spike-based encoding. To ensure a rigorous evaluation framework and prevent data leakage, we adopted a patient-specific (intra-patient) evaluation protocol consistent with the personalized wearable monitoring deployment scenario described in Section 1. For each patient record, the annotated beats were grouped by class (N, SVEB, VEB, F), and each class was independently split into 80% training and 20% testing partitions. This per-patient, per-class splitting ensures that the model is trained and evaluated on beats from the same individuals while maintaining the original class distribution within each partition.

#### 3.1.3. RR-Interval Feature Extraction

In addition to the morphological waveform features, four temporal features derived from *RR* intervals were computed for each beat to capture heart rate variability and rhythm regularity information. The RR interval represents the time elapsed between consecutive R-peaks and provides critical context for distinguishing arrhythmia types that may exhibit similar waveform morphologies but differ in their temporal characteristics. The four RR features were computed as follows:

${\mathrm{RR}}_{\mathrm{prev}}:$ The time interval between the current beat and the immediately preceding beat, calculated as the difference in sample indices divided by the sampling frequency. This feature captures the duration since the last heartbeat, which is shortened in premature beats and prolonged following compensatory pauses.

${\mathrm{RR}}_{\mathrm{next}}:$ The time interval between the current beat and the immediately following beat, computed analogously. This feature provides forward-looking temporal context and helps identify post-ectopic compensatory pauses.

${\mathrm{RR}}_{\mathrm{ratio}}:$ The ratio of ${\mathrm{RR}}_{\mathrm{prev}}$ to ${\mathrm{RR}}_{\mathrm{next}}$, calculated as shown in Equation (1a). This ratio quantifies the relative timing asymmetry around the current beat; a value near 1.0 indicates regular rhythm, while significant deviations suggest premature or delayed beats.

${\mathrm{RR}}_{\mathrm{diff}}:$ This is the arithmetic difference between ${\mathrm{RR}}_{\mathrm{prev}}$ and ${\mathrm{RR}}_{\mathrm{next}}$, Equation (1b), providing a signed measure of timing asymmetry. Positive values indicate the preceding interval was longer than the following interval, while negative values indicate the opposite.

(1a) $$\begin{array}{cc} RR_{\mathrm{ratio}} & =\frac{RR_{\mathrm{prev}}}{RR_{\mathrm{next}}} \end{array}$$

(1b) $$\begin{array}{cc} RR_{\mathrm{diff}} & =RR_{\mathrm{prev}}-RR_{\mathrm{next}} \end{array}$$

During preprocessing, the four RR-interval features are appended to each 180-sample waveform, producing a 184-dimensional feature vector per beat. Prior to model input, however, the RR features are separated from the morphological waveform: the 180-dimensional waveform segment is passed to the shared convolutional backbone, while the four RR features are held aside. After the backbone produces its 480-dimensional feature representation, the RR features are concatenated with this representation at the input of the designated classification head(s), according to the routing strategy being evaluated (see Section 3.3). This separation ensures that the backbone always operates on morphological features only, while RR-interval information is injected exclusively at the decision level. To prevent information leakage from the test set, the RR-interval features were normalized using the mean and standard deviation computed from the training set of each fold. These training-derived statistics were then applied to normalize the RR features of the corresponding test set, ensuring that no test-set distributional information influenced the model during training or evaluation.

### 3.2. Model Development

#### 3.2.1. Spiking Neuron Model

The fundamental computational unit in the QCSNN is the leaky integrate-and-fire (LIF) neuron. In continuous time, the membrane potential $U_{i}^{\left(l\right)}$ of neuron *i* in layer *l* evolves according to

(2) $$\tau_{\mathrm{mem}}\frac{dU_{i}^{\left(l\right)}}{dt}=-\left(U_{i}^{\left(l\right)}-U_{\mathrm{rest}}\right)+R\;I_{i}^{\left(l\right)},$$

where $U_{\mathrm{rest}}$ is the resting potential, $\tau_{\mathrm{mem}}$ is the membrane time constant, *R* is the input resistance, and $I_{i}^{\left(l\right)}$ is the input current. For implementation in a discrete-time simulation framework, this is approximated as a first-order recurrence:

(3) $$U_{i}^{\left(l\right)}\left[t\right]=\beta \;U_{i}^{\left(l\right)}\left[t-1\right]+\left(1-\beta \right)\;X_{i}^{\left(l\right)}\left[t\right]-S_{i}^{\left(l\right)}\left[t-1\right]\;U_{\mathrm{thr}},$$

where β∈[0,1] is the membrane potential decay factor (analogous to $e^{-\Delta t/\tau_{\mathrm{mem}}}$), $X_{i}^{\left(l\right)}\left[t\right]$ is the weighted input at timestep *t*, $U_{\mathrm{thr}}$ is the firing threshold, and $S_{i}^{\left(l\right)}\left[t-1\right]$ is the binary spike output from the previous timestep. A spike is emitted when the membrane potential reaches or exceeds the threshold:

(4) $$S_{i}^{\left(l\right)}\left[t\right]=\left\{\begin{array}{cc} 1, & \mathrm{if}\;U_{i}^{\left(l\right)}\left[t\right]\geq U_{\mathrm{thr}},\\ 0, & \mathrm{otherwise}. \end{array}\right.$$

Upon firing, the membrane potential is reset by subtracting the threshold value (subtract reset), as reflected in the last term of Equation (3). In this work, both β and $U_{\mathrm{thr}}$ are initialized to 0.5 and set as learnable parameters, allowing the network to adapt its temporal dynamics during training. The non-differentiable spike function in Equation (4) is approximated during backpropagation using a fast sigmoid surrogate gradient with slope parameter k=25 [21].

#### 3.2.2. Network Architecture

The QCSNN model employs a dual-head architecture consisting of a shared convolution backbone or main body followed by task-specific classification heads. As shown in Figure 2, the backbone network comprises three quantized convolution blocks, each containing a QuantConv1d layer, BatchNorm1dToQuantScaleBias for batch normalization, a leaky integrate-and-fire (LIF) spiking neuron, and MaxPool1d for spatial downsampling. The channel progression follows a 1→16→16→24 pattern across the three blocks, with each convolution layer using a kernel size of 3 and stride of 1. After flattening, the backbone produces a 480-dimensional feature representation that feeds into two task-specific quantized classification heads. The binary classification head distinguishes Normal from Abnormal heartbeats through a single quantized linear layer mapping 480 features to 2 output classes, followed by a LIF neuron. The multi-class classification head performs fine-grained 4-class arrhythmia classification (Normal, SVEB, VEB, Fusion) through two quantized linear layers—the first mapping 480 features to 128 hidden units with an intermediate LIF neuron, and the second mapping 128 units to 4 output classes followed by a final LIF neuron.

During training, both heads are optimized jointly using a combined loss function, enabling the shared backbone to learn feature representations beneficial for both classification tasks. All layers were quantized to 8-bit precision using the Brevitas [42] and snnTorch [41] quantization-aware training frameworks, with Int8WeightPerTensorFloat for weights and Int8ActPerTensorFloat for activations, enabling efficient deployment on FPGA hardware.

For inference, the architecture was converted into a two-stage cascaded QCSNN by disconnecting the multi-class head from the shared backbone and inserting a conditional routing mechanism after the binary head, as shown in Figure 3. In this cascaded configuration, the binary head serves as Stage-1, acting as a gating classifier that determines whether each input sample requires further analysis. When the binary head predicts Normal (class 0), the sample exits the pipeline immediately with a final classification of Normal, bypassing any additional computation. Conversely, when the binary head predicts Abnormal (class 1), the conditional routes the sample to Stage-2, where the multi-class head performs fine-grained classification into one of four arrhythmia categories: Normal, SVEB, VEB, or Fusion. This cascaded design offers two key advantages: first, it reduces computational overhead by processing only suspected abnormal heartbeats through the more complex multi-class classifier, and second, it enables Stage-2 to potentially self-correct Stage-1 misclassifications by predicting Normal for samples that were incorrectly flagged as Abnormal. The routing percentage—the proportion of samples forwarded to Stage-2—serves as an efficiency metric, with lower values indicating that more samples are resolved at Stage-1 without requiring additional processing.

### 3.3. Model Training and Evaluation

The training methodology employed stratified 6-fold cross-validation to ensure robust performance estimation and to mitigate overfitting, with each fold maintaining the original class distribution. The model was trained using the Adam optimizer with a learning rate of 0.0001 for 80 epochs, with a batch size of 128 and early stopping triggered after 10 epochs of no improvement (patience), with early stopping activation beginning after epoch 20. The spiking neural network was simulated over 10 timesteps, with spike outputs averaged across timesteps for classification. The LIF neurons were configured with an initial membrane potential decay factor (beta) of 0.5 and firing threshold of 0.5, both set as learnable parameters, and utilized the fast sigmoid surrogate gradient with a slope of 25 for backpropagation through the non-differentiable spike generation.

#### 3.3.1. Addressing Class Imbalance

The dataset exhibited substantial class imbalance, with the original distribution shown in Table 1. To address the adverse impact of this imbalance on model learning, particularly for minority classes with high clinical relevance, we explored a combination of choice of loss function and Synthetic Minority Over-sampling Technique (SMOTE) applied exclusively to the training set of each fold, thereby preventing data leakage while providing balanced training data.

The loss functions explored in each heads Cross-Entropy (CE) and Focal Loss (FL) [26]; shown in Table 2 and their representations are given in Equations (5) and (6):

(5a) $$L_{\mathrm{WCE}}=-\alpha_{\mathrm{CE}}\log\left(p_{\mathrm{CE}}\right)$$

(5b) $$\alpha_{\mathrm{CE}}=\frac{N}{C\cdot n_{\mathrm{CE}}}$$

(6a) $$L_{\mathrm{FL}}=-\alpha_{\mathrm{FL}}{\left(1-p_{\mathrm{FL}}\right)}^{\gamma }\log\left(p_{\mathrm{FL}}\right)$$

(6b) $$\alpha_{\mathrm{FL}}=\frac{N/(C\cdot n_{\mathrm{FL}})}{\sum_{j=1}^{C}N/\left(C\cdot n_{j}\right)}$$

where *p* is the predicted probability for the ground-truth class, α is the per-class weighting factor, *N* is the total number of samples, *C* is the number of classes, *n* is the number of samples in the ground-truth class, and γ≥0 is the focusing parameter that down-weights well-classified examples.

#### 3.3.2. Experimental Configurations and RR Feature Routing

To systematically investigate the interaction between loss function selection and RR-interval feature routing, we designed a factorial experiment comprising eight training configurations. These configurations span two loss functions (Cross-Entropy and Focal Loss) crossed with four RR-interval routing strategies, as summarized in Table 3.

Across all configurations, the shared convolutional backbone receives the 180-sample normalized morphological waveform and produces a 480-dimensional feature representation. The four RR-interval features (RR_prev_, RR_next_, RR_ratio_, RR_diff_) are not passed through the backbone; instead, they are concatenated with the backbone’s 480-dimensional output at the input of the designated classification head(s), yielding a 484-dimensional head input where applicable. The four routing strategies are defined as follows:

- **No RR:** Neither head receives RR features. Both the binary and multi-class heads operate on the 480-dimensional backbone output only. This serves as the baseline condition.
- **RR→S1:** Only the binary classification head (Stage-1) receives the concatenated 484-dimensional input (backbone features + RR). The multi-class head (Stage-2) receives the 480-dimensional backbone output only.
- **RR→S2:** Only the multi-class classification head (Stage-2) receives the concatenated 484-dimensional input. The binary head (Stage-1) operates on the 480-dimensional backbone output only.
- **RR→Both:** Both heads receive the 484-dimensional concatenated input. RR features are available to the entire inference pipeline.

This design isolates the RR feature routing from the shared feature extraction: the backbone learns morphological representations identically across all configurations, and only the head-level inputs vary. All other architectural parameters, training hyperparameters, and data splits remain identical across the eight configurations, ensuring that observed performance differences are attributable solely to the loss function and RR routing factors.

This factorial design enables two levels of analysis. First, it permits direct comparison of CE and FL under each routing condition, and of each routing strategy under each loss function. Second, and more importantly, it enables analysis of the *interaction* between these two factors—specifically, whether the inclusion of RR features modulates the relative advantage of Focal Loss over Cross-Entropy for minority-class arrhythmia detection.

The best-performing configuration was selected for FPGA deployment based on a combination of overall accuracy, macro-averaged F1 score, and minority-class (SVEB, VEB, F) recall, prioritizing balanced performance across all arrhythmia categories over raw accuracy alone.

Prior to training each fold, a Brevitas warmup procedure was executed to calibrate the quantization scaling factors by passing 10 batches of 8 samples through the network. Model selection within each fold was based on a combined score computed as a weighted sum of binary-class and multi-class performance metrics. Final evaluation utilized a two-stage cascaded inference pipeline, where the binary classifier’s argmax prediction determined routing, and comprehensive per-class metrics including precision, recall, specificity, and F1 score were computed for both stages.

Model training was conducted using the brevitas 0.12.1 [42] and snnTorch 0.9.1 [41] toolkits (Python 3.12.2; PyTorch, CUDA 12.4 build) on an NVIDIA RTX A2000 GPU (NVIDIA Corporation, Santa Clara, CA, USA). Each QCSNN architecture was trained independently using Quantization-Aware Training (QAT) with 8-bit integer weights under a symmetric quantization scheme to minimize memory and arithmetic overhead while maintaining classification accuracy. Training employed surrogate-gradient descent to overcome the non-differentiability of spiking activations, enabling end-to-end optimization within the spiking domain.

#### 3.3.3. Spike Encoding

SNNs require discrete spike trains as input rather than continuous-valued signals. In this work, we adopted the Direct Input Encoding approach [44], where the initial convolution block (i.e., the first quantized convolution, batchnorm, LIF and maxpool layers) of the network directly transforms the normalized ECG segments into spike representations. The LIF neurons integrate weighted inputs and emit spikes when membrane potentials exceed the trained threshold. The trained membrane leak dynamics serve as a temporal filtering mechanism, attenuating irrelevant or noisy fluctuations while preserving essential signal components and improving classification performance and ensuring a computationally efficient yet accurate transformation of raw ECG into spike-domain representations; thereby enabling real-time processing on constrained hardware platforms. This encoding scheme avoids the overhead of an explicit spike generation function and helps maintain sparsity in both convolution and dense layers.

### 3.4. FPGA Acceleration Methodology

The target deployment platform in this work is the PYNQ-Z2 development board (TUL Corporation, New Taipei City, Taiwan), which is built around the AMD-Xilinx Zynq-7020 system-on-chip (SoC; Advanced Micro Devices, Inc., Santa Clara, CA, USA) [45]. The Zynq-7020 integrates a dual-core ARM Cortex-A9 processing system (PS) with reconfigurable FPGA logic (the programmable logic, or PL), allowing the ARM cores to handle high-level orchestration in Python (PYNQ v3.0.1) while the FPGA fabric executes the quantized inference pipeline. We selected PYNQ-Z2 because it exposes the full Vivado/Vitis HLS (version 2025.1) design flow at an accessible price point and is widely available in academic and industrial settings, enabling third-party reproduction of the FPGA deployment results reported in Section 4.6. The gap between this development board and a production wearable target is acknowledged and discussed in Section 4.8 (Limitations and Future Directions).

**Figure 4:**
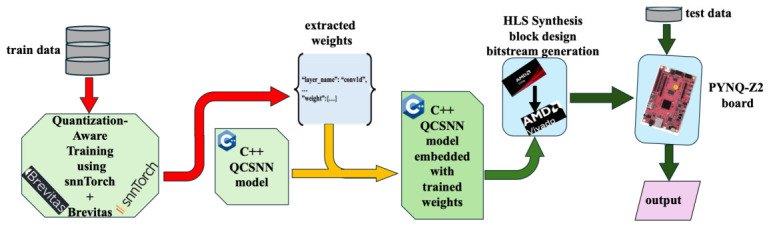
Illustration of the software–hardware integration workflow.

The complete flow of training, quantization, C++ model translation, and hardware deployment is depicted in Figure 4. After finalizing training, the quantized weights and network parameters were exported and integrated into a unified C/C++ implementation for FPGA synthesis.

The C/C++ implementation was synthesized using AMD’s Vitis High-Level Synthesis (HLS) toolchain, version 2025.1 [46], which automatically translated the software-based inference logic into a Register Transfer Level (RTL) hardware description. During HLS, optimization directives such as loop pipelining, resource sharing, and interface specification were applied to enhance performance and reduce hardware utilization. The resulting hardware description was encapsulated as a single reusable intellectual property (IP) core (topFunction) that internally implements both the binary (Stage-1) and four-class (Stage-2) classification paths.

**Figure 5:**
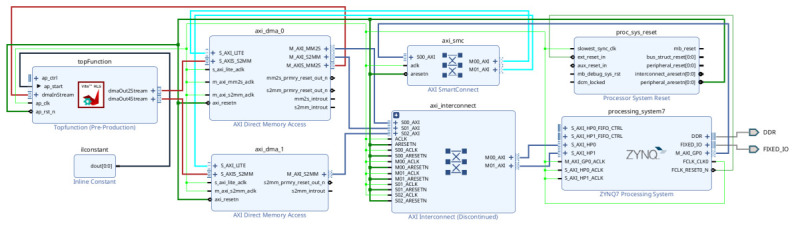
Top-level Vivado block design for the QCSNN hardware implementation on the PYNQ-Z2 board.

This IP core was subsequently integrated into a complete hardware system using the AMD Vivado Design Suite [47], as illustrated in Figure 5. The design comprises the topFunction IP core interfaced to the ZYNQ7 ARM processing system (PS) through two AXI DMA engines and an AXI SmartConnect/Interconnect fabric. The IP core receives ECG feature vectors via a single input stream (dmaInStream) and produces two output streams simultaneously: dmaOut2Stream carrying the Stage-1 binary prediction, and dmaOut4Stream carrying the Stage-2 four-class prediction. Both stages execute within a single inference pass through the IP core, and the entire system is loaded as a single bitstream—no partial reconfiguration is required.

Critically, the cascaded routing logic is implemented entirely within the topFunction IP core on the PL—not in software on the ARM PS. After Stage-1 produces a binary prediction, the IP core internally evaluates the result: if the prediction is Normal (class 0), the pipeline terminates early; if Abnormal (class 1), the IP core routes the sample to Stage-2 for four-class classification. The final prediction is then output via the appropriate DMA stream. This fully hardware-resident decision path eliminates the need for PS-mediated routing between stages, reducing inference latency and removing software-in-the-loop overhead. Because the majority of heartbeats in continuous monitoring are Normal, this hardware-level early termination means that most beats are classified using only the Stage-1 computation, yielding lower average latency and energy consumption than a fixed full-pipeline execution. This represents a key architectural improvement over our prior work [40], where independently trained stages required the PS to manage inter-stage data transfer and routing decisions. The self-contained hardware pipeline enables autonomous, real-time ECG processing while maintaining sub-watt accelerator power consumption, suitable for wearable or embedded monitoring systems.

#### Deployment Validation Metrics

To validate deployment fidelity, the FPGA implementation was compared against the GPU-based (NVIDIA RTX A2000) reference across two categories of metrics: classification performance and computational characteristics.

*Classification metrics.* Per-class precision, recall, and F1 score were computed on the held-out test set for both the GPU (PyTorch, CUDA 12.4 build; Brevitas 0.12.1) and FPGA implementations using identical input data and evaluation protocol. Any discrepancy between GPU and FPGA classification outputs indicates quantization or numerical fidelity loss introduced during the software-to-hardware translation.

*Computational metrics.* Four computational characteristics were measured for both platforms:

- **Inference latency:** The wall-clock time to classify a single ECG beat through the full two-stage pipeline. On the GPU, latency was measured using Python’s time.perf_counter() averaged over the test set. On the FPGA, latency was measured at runtime on the PYNQ-Z2 platform via Python timing instrumentation in the Jupyter Notebook 6.4.12 environment, encompassing data transfer, accelerator execution, and result retrieval. R-peak detection is treated as an upstream preprocessing step performed prior to beat segmentation and is not included within the scope of the inference-latency metric.
- **Throughput:** The number of ECG beats classified per second, computed as the inverse of the average per-beat latency for each platform.
- **Power consumption:** GPU power was recorded using nvidia-smi during sustained inference. FPGA power was measured externally using an FNRSI^®^ FNB58 USB power tester, which captured total board power during inference. Component-wise power breakdown (accelerator logic, ARM PS, and static leakage) was obtained from the Vivado post-implementation power report.
- **Resource utilization:** For the FPGA, the consumption of look-up tables (LUTs), flip-flops (FFs), digital signal processing slices (DSPs), and block RAM (BRAM) was reported from the Vivado post-implementation utilization summary as a percentage of the PYNQ-Z2’s available resources. For the GPU, peak memory usage during inference was recorded.

Together, these metrics provide a comprehensive comparison of whether the FPGA deployment preserves the classification performance of the GPU baseline while achieving the latency, throughput, and power characteristics required for real-time wearable cardiac monitoring.

## 4. Results and Discussion

This section presents the results of the eight-configuration factorial experiment described in Section 3, evaluating the interaction between loss function selection (CE vs. FL) and RR-interval feature routing (No RR, RR→S1, RR→S2, R→Both) on the dual-head QCSNN. We begin with cross-validation stability analysis (Section 4.1), which establishes that single-fold reporting in the remainder of the section is representative of each configuration’s behavior. We then establish a baseline comparison of CE and FL without RR features (Section 4.2), then examine the effect of RR routing on per-stage performance (Section 4.3), followed by a dedicated analysis of the loss–RR-interaction (Section 4.4). Finally, we present the model selection rationale for FPGA deployment (Section 4.5). Statistical significance testing for the principal comparisons is reported alongside the corresponding analyses in Section 4.3 and Section 4.4. All models were evaluated on the held-out test set from the MIT-BIH Arrhythmia Database using per-class precision, recall (sensitivity), and F1 score.

### 4.1. Cross-Validation Stability

Before presenting per-class results, we first characterize the cross-fold stability of each configuration. Each of the eight configurations was trained under a six-fold stratified cross-validation protocol within the training subset (Section 3.3). For each fold, we evaluated aggregate metrics on the held-out test set; the fold selected for FPGA deployment was chosen by validation score on the training subset, and all subsequent per-class tables (Section 4.2, Section 4.3, Section 4.4 and Section 4.5) report results from this deployment fold. The cross-fold statistics reported in this subsection establish that the deployment-fold results are representative rather than outliers.

Table 4 reports the mean and standard deviation of aggregate metrics across the six folds for each of the eight configurations. Standard deviations are narrow throughout: Stage-2 macro F1 standard deviations range from 0.006 to 0.017 across all configurations, indicating that single-fold performance is highly representative of each configuration’s behavior. The routed percentage (proportion of beats forwarded from Stage-1 to Stage-2) is also stable across folds, with standard deviations below 1.6 percentage points for all configurations except CE:RR→S2 (1.55 pp). Statistical significance testing for the principal comparisons of interest is reported alongside the corresponding analyses in Section 4.3 and Section 4.4.

Given this cross-fold consistency, the per-class analyses that follow are reported from the FPGA-deployment fold of each configuration, with the understanding that fold-to-fold variability is small enough that the deployed model’s per-class behavior is representative of the configuration as a whole. For the deployment configuration (CE:RR→Both), the per-class precision, recall, and F1 across all six folds (mean ± standard deviation) are tabulated in Table S2 of the Supplementary Materials.

### 4.2. Baseline Model Performance

**Figure 6:**
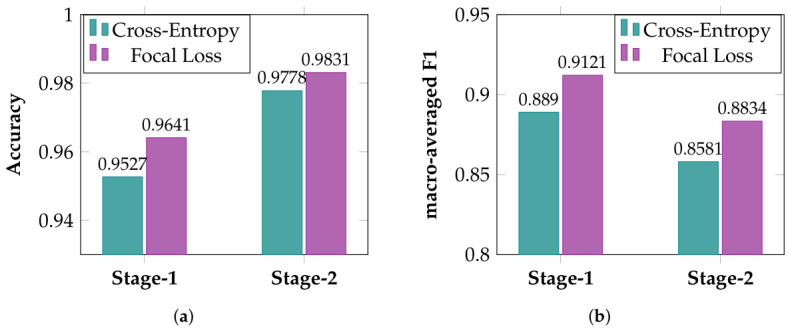
Stage-1 and Stage-2 performance comparison between Cross-Entropy and Focal Loss: (**a**) Accuracy; (**b**) macro-averaged F1.

Table 5 and Table 6 present the per-class performance of the baseline QCSNN (no RR features) under CE and FL training. Figure 6a,b summarize the stage-level accuracy and macro-averaged F1 for both configurations. In Stage-1, both loss functions achieve high Normal precision (∼99.2%), but FL yields notably better Abnormal precision (77.28% vs. 70.84%), reducing false positive routing to Stage-2 without sacrificing Abnormal recall (∼93% for both). This translates to a 2.31% F1 advantage for FL (91.21% vs. 88.90%).

In Stage-2, FL maintains a consistent edge across all four classes. The most pronounced gains appear in the minority classes: SVEB precision improves by 4.58% (86.52% vs. 81.94%), VEB precision by 2.93% (97.12% vs. 94.19%), and Fusion precision by 10.05% (67.66% vs. 57.61%). Recall values remain comparable between the two loss functions across all classes. The overall Stage-2 macro F1 advantage for FL is 2.53% (88.34% vs. 85.81%). These baseline results confirm the expected advantage of focal loss under class imbalance when the model operates on morphological features alone: FL improves minority-class precision without degrading recall. This FL–CE performance gap serves as the reference against which the effect of RR-interval features is evaluated in the following sections.

### 4.3. Effect of RR-Interval Features on Model Performance

**Figure 7:**
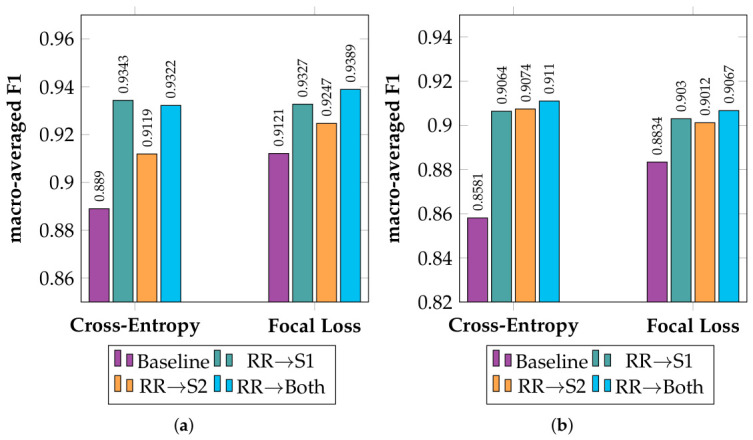
Macro-averaged F1 comparison across RR-interval routing configurations: (**a**) Stage-1; (**b**) Stage-2.

Having established that FL outperforms CE in the absence of RR features, we now examine how the introduction of RR-interval features under the four routing strategies defined in Section 3.3 affects this relationship. Figure 7a,b show the macro-averaged F1 for Stage-1 and Stage-2, respectively, across all eight configurations. A consistent pattern emerges: CE models benefited substantially more from the addition of RR features than FL models. For example, adding RR features to Stage-1 only (RR→S1) improved the CE model’s macro F1 from 88.90% to 93.43% in Stage-1 and from 85.81% to 90.64% in Stage-2—an approximate 5% increase in both stages, compared to only ∼2% for the FL model under the same routing condition.

#### 4.3.1. RR Features Effects on Stage-1

**Figure 8:**
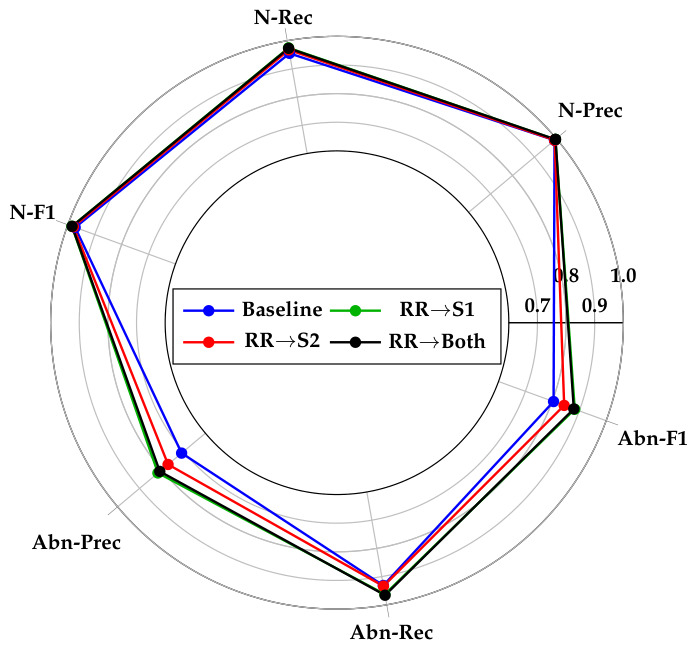
Stage-1 CE per-class performance comparison across RR routing configurations.

Table 7 displays and compares the effect of RR features on per-class metrics for Stage-1 for both CE and FL models. Table 7 shows that adding RR-interval features benefited CE models more than FL models. For example, under the RR→S1 configuration, Abnormal precision increased by 10.79% for the CE model compared to 4.26% for FL. RR features provide the discriminative temporal information that CE was previously lacking, substantially closing the performance gap with FL. The mechanistic explanation for this interaction is examined in Section 4.3. With RR features, as shown in Figure 8, the CE model achieves slightly better Abnormal recall (96.30% vs. 95.73%), meaning fewer missed arrhythmias enter Stage-2. This advantage increases further under RR→Both (96.63% vs. 95.50%), making it the best configuration for minimizing missed arrhythmias. However, under the RR→S2 configuration, the FL model significantly outperforms the CE model on Abnormal precision by 4.14%—FL achieves 81.15% precision against CE’s 77.01% for CE model. This means FL model produces fewer false positives when predicting Abnormal. This pattern mirrors the baseline performance. Although the CE model benefits more from RR→S2—consistent with the earlier observation—the FL model continues to achieve higher Abnormal precision. A recall trade-off emerges: the FL model favors Normal detection (97.48% vs. 96.74%), while the CE model favors Abnormal detection (93.41% vs. 92.89%).

#### 4.3.2. RR Features Effects on Stage-2

**Figure 9:**
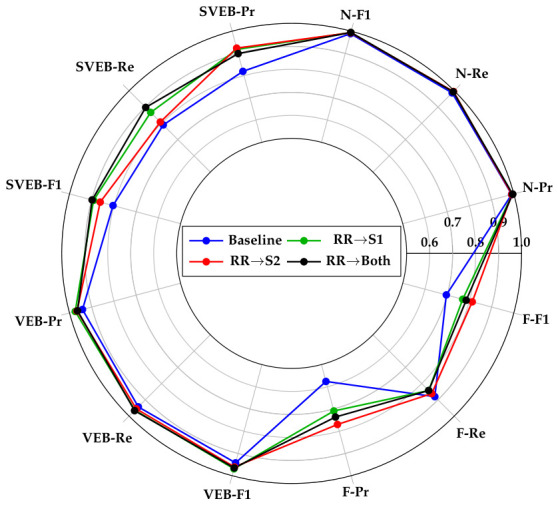
Stage-2 CE per-class performance comparison across RR routing configurations.

Table 8 presents the per-class effect of RR features on Stage-2 performance. Consistent with the Stage-1 findings, the CE model leverages RR features more effectively for precision across most classes. Under the RR→S1 configuration, the CE model’s precision shows significant gains over the baseline: +0.29% for N, +9.89% for SVEB, +3.27% for VEB, and +13.29% for F. Figure 9 depicts these CE-model performance improvements as a radar chart. The FL model’s precision also improved, though less markedly: +0.22% for N, +4.57% for SVEB, and +5.56% for F, while VEB precision degraded slightly by −0.82%. For recall, the CE model demonstrates higher sensitivity, detecting 86.71% of SVEB compared to FL’s 84.38%, and 96.41% of VEB compared to FL’s 95.41%. Both models achieve comparable recall for Normal (∼99.45%) and Fusion (∼84.28%). And for the instance RR→Both, CE model recall further improved from baseline values:

- N: 98.71%→99.37% (+0.66)
- SVEB: 78.99%→89.77% (+10.78)
- VEB: 94.33%→96.63% (+2.30)

The F class was however slightly degraded from 88.05% to 84.28% (−3.77). The RR→S2 configuration provided no clear advantage for either CE or FL models.

To formally assess whether the RR-driven improvements observed above are statistically significant, we applied the paired Wilcoxon signed-rank test (exact method, n=6 folds) comparing each RR-augmented configuration against its no-RR baseline on Stage-2 macro F1. With n=6, the normality assumption underlying a paired *t*-test cannot be verified; we use the Wilcoxon signed-rank test, which trades minor power for distributional robustness. Exact-method *p*-values are reported throughout; negative findings are interpreted with the small-*n* power limit in mind. With n=6 paired samples, the smallest achievable one-sided *p*-value is 0.016. Table 9 reports the test results. Under both Cross-Entropy and Focal Loss, the RR→S1 and RR→Both routings yield improvements that are significant at p=0.016 (the minimum achievable with n=6). The RR→S2 routing does not reach statistical significance (p=0.078 for CE; p=0.219 for FL). This pattern is consistent with the per-class evidence above: RR features contribute most when they inform Stage-1’s routing decision (RR→S1 or RR→Both) rather than only enriching Stage-2’s classification input (RR→S2 alone).

### 4.4. Loss Function–RR Feature Interaction Analysis

One of the central findings of this study deserves to be stated up front: simply adding RR-interval features at the input level provides more value for arrhythmia detection than tuning the loss function. Quantitatively, at the strongest routing (RR→Both), adding RR features lifts Stage-2 macro F1 by +0.034 under Cross-Entropy and +0.028 under Focal Loss (Table 9)—gains that both individually exceed the Focal-Loss-over-Cross-Entropy advantage of +0.013 in the no-RR baseline (Table 10). Moreover, this FL-over-CE advantage is no longer statistically detectable once RR features are added at any routing (Wilcoxon two-sided p≥0.219). The conventional logic of using focal loss to handle class imbalance is, in our setting, largely disrupted once temporal RR context is supplied—a shift in design priority from the optimization stage to the feature stage. This finding is observed for this dataset, with this architecture, at 8-bit (INT8) quantization; the boundary of the claim is set by these conditions.

The per-stage results presented above reveal a consistent pattern that transcends individual class metrics: the relative advantage of Focal Loss over Cross-Entropy is strongly modulated by the presence or absence of RR-interval features. Table 11 quantifies this interaction by showing the FL–CE performance gap (measured as ΔF1 = FL_F1_− CE_F1_) across all routing configurations for both stages.

Two findings emerge from this analysis. First, in the baseline condition (no RR features), FL consistently outperforms CE by approximately 2.3–2.5% F1 in both stages. This confirms the expected advantage of focal loss under class imbalance when the model relies on morphological features alone. Second, and more importantly, when RR-interval features are introduced under any routing strategy, this gap narrows dramatically and in several configurations reverses entirely, with CE matching or surpassing FL. In Stage-2—where the clinically critical multi-class discrimination occurs—CE outperforms FL under all three RR-augmented conditions.

This interaction effect suggests that FL and RR-interval features address minority-class underperformance through partially overlapping mechanisms. Without RR features, the model’s input representation provides limited discriminative power for rare arrhythmia classes, and FL compensates by reshaping the loss landscape to prioritize these difficult samples. When RR features are introduced, they supply explicit temporal context—beat prematurity, compensatory pauses, rhythm regularity—that directly disambiguates the morphologically similar beats that CE previously struggled with. In effect, RR features provide at the input level much of the discriminative information that FL was recovering at the optimization level, reducing the marginal benefit of focal loss’s reweighting mechanism.

The collapse of the FL–CE gap with the introduction of RR features is supported by paired Wilcoxon signed-rank testing (two-sided, n=6 folds; smallest achievable two-sided p≈0.031). Table 10 reports the FL-vs-CE comparison at each routing configuration. Without RR features, FL significantly outperforms CE on Stage-2 macro F1 (Δ=+0.013, p=0.031). Once RR features are introduced under any routing strategy, the FL advantage is no longer statistically detectable (p≥0.219 for all three RR-augmented routings). This pattern—a statistically significant FL advantage that disappears when RR features are added—directly mirrors the practical-magnitude finding shown in Table 11 above and provides formal support for the interpretation that RR-interval features at the input level supplant the optimization-level benefit that Focal Loss provides under class imbalance.

This finding has practical implications for embedded deployment. Cross-entropy loss is simpler to implement and tune than focal loss, which requires careful selection of the focusing parameter γ and class-specific α weights. If RR features are available—as they are in any system that performs R-peak detection, a prerequisite for beat segmentation—then CE combined with RR features offers a simpler training pipeline with equal or better classification performance compared to FL. This favors CE:RR→Both as the preferred configuration for hardware deployment, a selection further justified in the following section.

### 4.5. Model Selection

To select the model configuration for FPGA deployment, we defined selection criteria informed by clinical requirements for wearable continuous cardiac monitoring. Table 12 summarizes these criteria and their priority levels, drawn from established guidelines and prior deployment studies. The two critical requirements are high Abnormal recall in Stage-1 (to avoid missed arrhythmias) and high VEB precision and recall in Stage-2 (given the clinical severity of ventricular ectopic beats). Based on these criteria, CE:RR→Both emerged as the preferred configuration. Its key advantages are summarized alongside the full per-stage metrics in Table 13. In Stage-1, this configuration achieves the highest Abnormal recall (96.63%) across all eight configurations, minimizing the risk of missed arrhythmias entering Stage-2. In Stage-2, it delivers the strongest SVEB recall (89.77%) and VEB recall (96.63%) while maintaining the highest macro-averaged F1 (91.10%). Although its Abnormal precision (80.78%) is approximately 3% lower than FL:RR→Both (83.60%), this marginal increase in false alarms represents an acceptable trade-off: in a wearable deployment where user safety is paramount, a false alarm is an inconvenience, while a missed arrhythmia is a safety failure.

An additional practical advantage, as discussed in Section 4.3, is that CE:RR→Both employs the simpler cross-entropy loss function, avoiding the hyperparameter sensitivity of focal loss (γ, per-class α) and simplifying the training pipeline for deployment-oriented development.

### 4.6. FPGA Acceleration Results

The preceding sections evaluated all eight configurations on the GPU (NVIDIA RTX A2000) and selected CE:RR→Both for hardware deployment. This section presents the deployment of this configuration on the PYNQ-Z2 FPGA and validates its operational fidelity following the evaluation framework defined in Section 3.4. We compare GPU and FPGA implementations across classification metrics, inference latency, throughput, power consumption, and resource utilization to determine whether the software-to-hardware translation preserves diagnostic performance while meeting the computational constraints of edge deployment.

#### 4.6.1. GPU vs. FPGA Inference Performance

**Figure 10:**
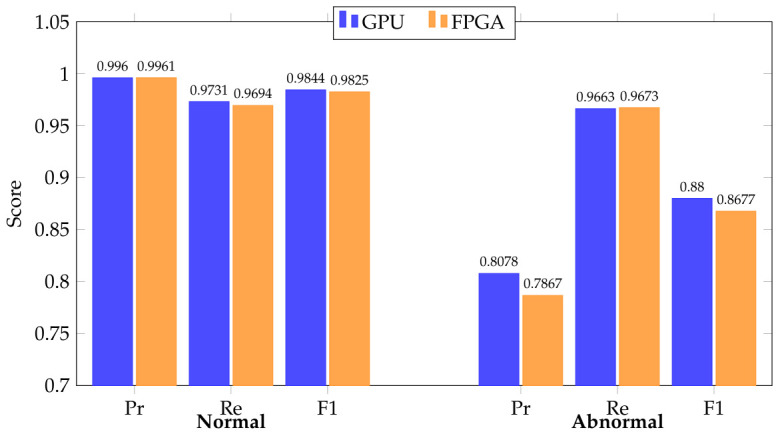
QCSNN Stage-1: GPU vs FPGA per-class performance comparison.

**Figure 11:**
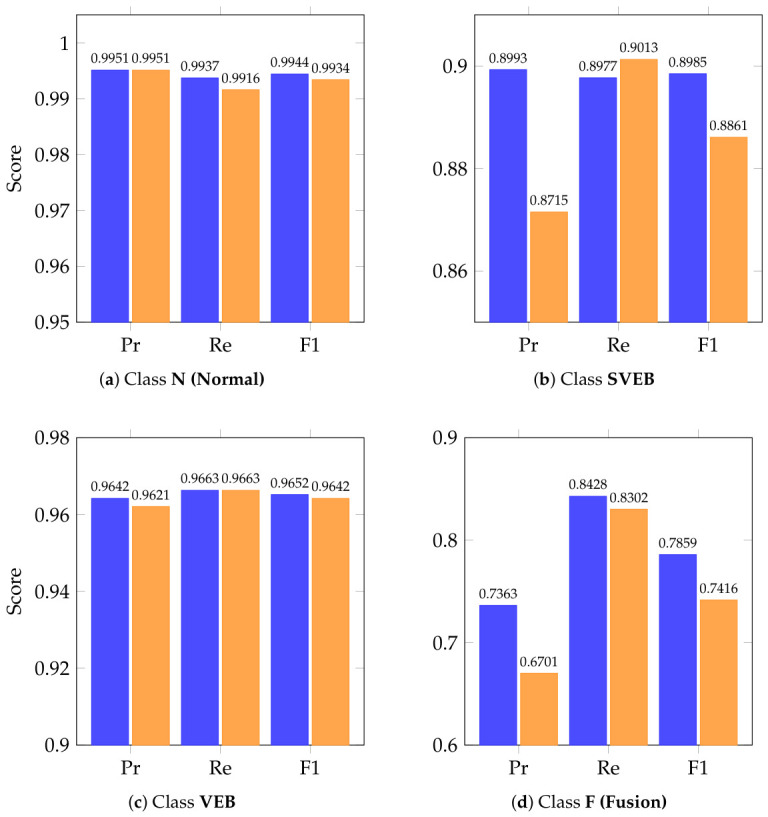
QCSNN Stage-2: GPU vs FPGA per-class performance comparison: (**a**) N (Normal); (**b**) SVEB; (**c**) VEB; (**d**) F (Fusion).

To evaluate deployment fidelity, the trained CE:RR→Both model was translated to C/C++ [57] and deployed on the PYNQ-Z2 FPGA. The FPGA implementation was evaluated on the identical held-out test set used for GPU evaluation. Table 14 reports the FPGA classification metrics, while Figure 10 and Figure 11 provide a side-by-side GPU–FPGA comparison for both stages. *Stage-1.* The FPGA model closely matches the GPU reference across all Stage-1 metrics. Normal precision is identical (99.60%), with recall and F1 within 0.4% and 0.2%, respectively, (FPGA: 96.94%, 98.25% vs. GPU: 97.31%, 98.44%). For the Abnormal class, the FPGA achieves marginally higher recall than the GPU (96.73% vs. 96.63%), confirming that arrhythmia detection sensitivity is preserved after hardware translation. Abnormal precision is lower on the FPGA (78.67% vs. 80.78%), likely attributable to rounding differences in fixed-point accumulation between the GPU and FPGA inference pipelines, despite both using identical INT8 quantized weights and architectural structures. *Stage-2.* The clinically critical classes maintain strong fidelity on FPGA. VEB recall is identical across platforms (96.63%), with precision and F1 within 0.2% and 0.1% respectively (FPGA: 96.21%, 96.42% vs. GPU: 96.42%, 96.52%). SVEB recall is marginally higher on FPGA (90.13% vs. 89.77%), while precision and F1 show modest degradation (FPGA: 87.15%, 88.61% vs. GPU: 89.93%, 89.85%). The Fusion class exhibits the largest discrepancy, with FPGA precision dropping to 67.01% compared to GPU’s 73.63%—consistent with this class’s inherent ambiguity and sensitivity to numerical precision. Normal class performance is virtually identical across both platforms. Overall, the FPGA implementation preserves the GPU’s diagnostic sensitivity for the two most clinically important arrhythmia classes (VEB and SVEB), with minor precision degradation concentrated in the Abnormal and Fusion classes. These results validate that the software-to-hardware translation introduces no clinically significant performance loss, confirming the QCSNN’s suitability for edge-deployed continuous cardiac monitoring.

#### 4.6.2. Resource Utilization on PYNQ-Z2

Table 15 details the hardware resource utilization of the QCSNN deployed on the PYNQ-Z2. The model saturates all available DSP slices (220 out of 220), indicating full utilization of the FPGA’s arithmetic processing capacity. Look-Up Table (LUT) utilization reached 36.80%, and Block RAM (BRAM) usage was 67.14%, both remaining within comfortable operating margins. These figures reflect the inference-optimized nature of the architecture, where performance is primarily bottlenecked by DSP availability. The full DSP saturation indicates that the current design would not accommodate additional computational layers without architectural optimization, a consideration discussed further in Section 5.

#### 4.6.3. Power Consumption and Latency Analysis

**Figure 12:**
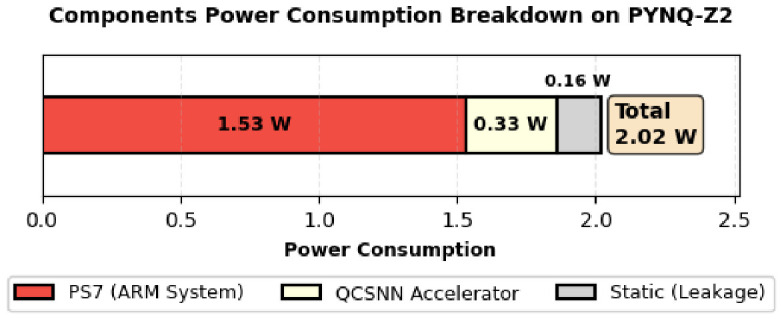
Component-level power consumption breakdown of the PYNQ-Z2 system, showing the contribution of the QCSNN accelerator relative to the ARM processing system and static power.

Power and latency analysis further demonstrate the FPGA’s suitability for edge deployment. Figure 12 shows the component-wise power breakdown on the PYNQ-Z2 board, where the QCSNN accelerator consumes only 0.33 W out of a total system power of 2.02 W. The remaining power is attributed to the ARM PS and leakage. Measurements were validated using the FNRSI^®^ USB Fast Charge Tester FNB58.

Table 16 compares the energy efficiency and inference speed of the FPGA and a desktop-class RTX A2000 GPU. While the GPU offers 7.89× higher throughput (683.72 vs. 86.63 samples/s) and 2.8× lower energy per inference (23.305 mJ vs. 93.55 mJ), it also consumes 31.66× more power (63.96 W vs. 2.02 W). The reported 11.54 ms per-beat latency reflects the classification-pipeline time only, consistent with the scope of the inference-latency metric defined in Section 3.4: R-peak detection, performed prior to beat segmentation, is not included in this figure. For reference, lightweight R-peak detectors such as Pan–Tompkins [58] add only a few milliseconds per beat on embedded ARM processors—their accuracy on low-quality ECG signals has been well characterized in the literature [59]—so end-to-end beat-to-decision latency including R-peak detection remains well inside the 500–1000 ms per-beat budget of real-time continuous ECG monitoring. For *continuous, real-time ECG monitoring applications*, where inference occurs every 500–1000 ms, the PYNQ-Z2’s 11.54 ms latency and sub-watt power consumption make it far more viable for deployment in smart wearables, medical patches, or home monitoring devices. The GPU’s high throughput is more suited to batch inference or cloud-based analytics but is overkill for beat-by-beat streaming analysis.

Table 17 consolidates the deployment validation results across all metrics defined in Section 3.4. The FPGA implementation preserves classification fidelity—with Stage-1 and Stage-2 macro-averaged F1 within 1.0% and 0.7% of the GPU reference, respectively—while delivering 31.66× lower power consumption and 4.01× lower energy per inference. Although the GPU achieves 7.89× higher throughput, the FPGA’s 86.63 samples/s (11.54 ms per beat) comfortably exceeds the requirements of real-time beat-by-beat monitoring, where inter-beat intervals typically range from 500–1000 ms. These results confirm that the software-to-hardware translation preserves diagnostic performance within clinically acceptable margins while meeting the power and latency constraints of wearable edge deployment.

### 4.7. Comparison with Prior Work

Table 18 and Table 19 position the QCSNN against recent arrhythmia classification models evaluated on the MIT-BIH database. Table 18 compares model architecture and classification performance, while Table 19 focuses on hardware-deployed implementations. Direct accuracy comparison requires caution, as evaluation protocols differ across studies; in particular, intra-patient protocols typically yield higher accuracy than the inter-patient AAMI protocol due to shared patient morphology between training and test sets.

With this separation, the QCSNN’s positioning becomes clear across both dimensions. In Table 18, the QCSNN achieves the highest accuracy among all SNN-based models (99.02%) while using 6.6× fewer parameters than the nearest SNN competitor (SNN+CAM: 435,336) and up to 9.7× fewer than the largest DNN (CNN-BiLSTM: 634,621). At 0.064 MB (INT8), it is the smallest deployed model in the comparison.

In Table 19, the QCSNN is the only model combining SNN computation, full INT8 quantization-aware training, and validated PYNQ-Z2 deployment. Compared to the only other PYNQ-Z2 model (PCGQ-CNN), the QCSNN achieves higher accuracy (99.02% vs. 97.98%) with 3.9× lower latency (11.54 ms vs. 45.1 ms). The Scrugli et al. [62] All-Spiking SNN achieves substantially lower power (11.8 mW) and energy (50.98 μJ) on a Lattice iCE40 FPGA—a much smaller and lower-power device than the Zynq-7020 on the PYNQ-Z2, making direct power comparison inappropriate. The Chu et al. [6] spike-driven processor achieves remarkable energy efficiency (0.75 μJ) through custom 40 nm ASIC design—a fundamentally different deployment target. A distinguishing aspect of the QCSNN is the combination of SNN efficiency, INT8 quantization, two-stage cascaded inference with hardware-level early termination, and FPGA deployability in a unified framework—a combination not reported, to our knowledge, in prior PYNQ-Z2 deployments of directly trained SNNs.

### 4.8. Limitations and Future Directions

Several limitations of the present work warrant explicit discussion. The primary results use a patient-specific (intra-patient) protocol, motivated by the target deployment scenario of continuous personalized cardiac monitoring on a wearable device (Section 1); inter-patient generalization is therefore not claimed and is explicitly outside the scope of this work. To characterize the model’s transferability beyond the patient-specific scenario, we conducted an exploratory inter-patient evaluation under the standard AAMI DS1/DS2 protocol [9] (a full 2 × 2 CE/FL × Baseline/RR→Both ablation, with six-fold cross-validation within DS1 and final evaluation on the held-out DS2 records); the deployment configuration (CE:RR→Both) achieved an overall Stage-2 accuracy of 89.31 ± 1.51% on DS2, competitive with recent inter-patient classifiers (Sellami and Hwang [64], 88.34%; Mondéjar-Guerra et al. [65], 94.5%) on the same split, and the central interaction finding of the intra-patient study is preserved under cross-patient distribution shift (the Focal-Loss-over-Cross-Entropy Stage-2 accuracy advantage shrinks from +2.67 pp without RR features to +0.93 pp once RR features are added, while the input-level RR-interval contribution itself transfers, with Stage-1 and Stage-2 macro F1 gains of ≈+0.115 and ≈+0.066 respectively, and a Stage-2 accuracy gain of ≈+6.2 pp, over the no-RR baseline); class-by-class, the Normal and VEB classes generalize comparably across all three methods (our Normal F1 = 0.946 vs. 0.934/0.970; our VEB F1 = 0.804 vs. 0.809/0.943), while minority-class (SVEB and Fusion) performance is lower in our model and is identified as an open research direction. The ≈5 pp accuracy gap between our 89.31% and Mondéjar-Guerra et al.’s 94.5% reflects a substantive design trade-off rather than a deficit—handcrafted multi-resolution features and full-precision arithmetic versus four RR-interval features alongside the raw waveform at 8-bit (INT8) quantization—and a comparable feature-engineering ablation under full precision, together with an inter-patient evaluation of richer handcrafted feature sets within the SNN+FPGA pipeline, is identified as future work. We emphasize that these inter-patient results are exploratory; the intra-patient personalized-deployment evaluation remains the methodologically appropriate measure for the target use case, and comprehensive inter-patient generalization is identified as future work in Section 5. Beyond this evaluation-protocol limitation, four practical limitations also warrant note: (i) the Fusion (F) class consistently exhibits the lowest precision and the largest GPU–FPGA fidelity gap across our experiments, reflecting both its under-representation in the dataset and its hybrid supraventricular–ventricular morphology, and characterizing and mitigating this gap under quantized deployment is an open future-work item; (ii) the PYNQ-Z2 was selected to enable a full FPGA design flow (Vitis HLS → Vivado → PYNQ overlay) on widely-accessible, reproducible hardware but, as a development board, provides a larger silicon and power envelope than a production wearable would target, so migration to a deployment-grade target is identified as future work; (iii) the MIT-BIH database, while the standard benchmark, was acquired several decades ago and the present evaluation uses only the single MLII lead, so validation on more recent multi-lead datasets and on ambulatory data from commercial wearables remains an important future step; and (iv) the classifier’s accuracy is conditional on accurate upstream R-peak detection—under degraded signal quality (motion artifact, muscle noise, transient electrode loss) R-peak-detection errors will propagate into the classifier and degrade downstream performance, so joint R-peak/classification training on noisy wearable signals and a quantitative characterization of R-peak-detection robustness on commercial wearable ECG signals are also targets for future work.

## 5. Conclusions

This work demonstrates that accurate and efficient edge-based arrhythmia detection can be achieved with a directly trained, quantized SNN. Beyond introducing the QCSNN framework, the central finding is that RR-interval features substantially diminish the benefit of focal-loss-based imbalance correction: Focal Loss improves macro F1 when RR features are absent, but this advantage largely disappears once RR features are included, with Cross-Entropy matching or exceeding its performance while offering a simpler training pipeline. This suggests that temporal RR information provides a discriminative context that alleviates part of the imbalance challenge at the input level. Together with jointly trained dual-head learning and cascaded inference, these findings make the proposed framework especially attractive for wearable deployment. The FPGA results further show that these gains can be realized on resource-constrained hardware with low latency and low power, without clinically meaningful degradation in diagnostic performance. Quantitatively, the central interaction finding can be summarized as follows: at the strongest routing, adding RR-interval features lifts Stage-2 macro F1 by +0.028 to +0.034 depending on loss function, both gains exceeding the entire +0.013 Focal-Loss-over-Cross-Entropy advantage in the no-RR baseline—an advantage that is no longer statistically detectable once RR features are added at any routing. The optimization-level fix is supplanted by an input-level fix. Overall, the study establishes quantized SNNs as a practical foundation for real-time, on-device arrhythmia monitoring. Beyond the loss-function and feature-interaction findings, the practical contribution of this work is to demonstrate that multi-class arrhythmia inference at clinically meaningful accuracy can be sustained directly on the wearable, decoupling the device from the cloud entirely and preserving function during connectivity loss, without the continuous-transmission energy burden of cloud-centric pipelines. Future work will focus on improving hardware efficiency through lower-precision and sparsity-aware implementations, reducing precision loss in challenging classes such as Fusion and evaluating generalization under inter-patient protocols.

## Figures and Tables

**Table 1:** ECG beat distribution.

	Normal	SVEB	VEB	F
Original	72,073	5616	2224	644
After SMOTE	72,073	72,073	72,073	72,073

**Table 2:** Loss functions used per classification head.

Head	Loss Function	
binary-class	CE + cw	FL + α
multi-class	CE	FL

**Table 3:** Eight experimental configurations defined by the factorial combination of loss function and RR-interval routing strategy. The shared backbone always receives the 180-dimensional morphological waveform. RR features are concatenated at the classification head level only.

ID	Configuration	Stage-1 Head	Stage-2 Head
1	CE:No RR	480	480
2	CE:RR→S1	480 + 4	480
3	CE:RR→S2	480	480 + 4
4	CE:RR→Both	480 + 4	480 + 4
5	FL:No RR	480	480
6	FL:RR→S1	480 + 4	480
7	FL:RR→S2	480	480 + 4
8	FL:RR→Both	480 + 4	480 + 4

**Table 4:** Aggregate performance metrics across 6 cross-validation folds for each of the eight training configurations. Values are mean ± standard deviation. The combined score is a balanced metric used for model selection (see Section 3.3). The Stage-1 column reports F_2_-score for the abnormal class (recall-weighted, β=2), which prioritizes arrhythmia-detection sensitivity in line with the clinical *don’t miss arrhythmias* criterion that governs Stage-1 model selection (see Section 4.5).

Configuration	Routed %	Stage-1 Acc (%)	Stage-1 F_2,abn_	Stage-2 Acc (%)	Stage-2 F1_macro_	Combined
CE:No RR	13.51 ± 0.58	95.32 ± 0.58	0.870 ± 0.011	98.00 ± 0.10	0.877 ± 0.006	0.872 ± 0.008
CE:RR→S1	12.51 ± 0.17	97.26 ± 0.09	0.929 ± 0.004	98.63 ± 0.18	0.906 ± 0.012	0.922 ± 0.006
CE:RR→S2	13.72 ± 1.55	95.18 ± 1.56	0.870 ± 0.028	98.32 ± 0.31	0.890 ± 0.017	0.876 ± 0.024
CE:RR→Both	12.62 ± 0.33	97.14 ± 0.36	0.927 ± 0.008	98.62 ± 0.25	0.911 ± 0.016	0.922 ± 0.010
FL:No RR	12.03 ± 0.31	96.67 ± 0.25	0.888 ± 0.008	98.29 ± 0.17	0.890 ± 0.012	0.888 ± 0.009
FL:RR→S1	11.95 ± 0.18	97.61 ± 0.29	0.929 ± 0.010	98.64 ± 0.14	0.906 ± 0.013	0.922 ± 0.010
FL:RR→S2	12.02 ± 0.31	96.58 ± 0.44	0.883 ± 0.013	98.42 ± 0.24	0.900 ± 0.015	0.888 ± 0.013
FL:RR→Both	12.10 ± 0.30	97.52 ± 0.26	0.929 ± 0.005	98.70 ± 0.15	0.918 ± 0.010	0.926 ± 0.006

**Table 5:** Stage-1 binary classification performance.

	Cross-Entropy		Focal Loss	
	Normal	Abnormal	Normal	Abnormal
**Pr**	0.9917	0.7084	0.9917	0.7728
**Re**	0.9552	0.9317	0.9680	0.9308
**F1**	0.9731	0.8048	0.9797	0.8445

**Table 6:** Stage-2 four-class classification performance.

	Cross-Entropy			Focal Loss		
	Pr	Re	F1	Pr	Re	F1
**N**	0.9908	0.9871	0.9890	0.9907	0.9930	0.9919
**SVEB**	0.8194	0.7899	0.8044	0.8652	0.7953	0.8288
**VEB**	0.9419	0.9433	0.9426	0.9712	0.9440	0.9574
**F**	0.5761	0.8805	0.6965	0.6766	0.8553	0.7556

**Table 7:** Stage-1 binary classification performance across all configurations.

		Normal			Abnormal		
		Pr	Re	F1	Pr	Re	F1
**CE**	**Baseline**	0.9917	0.9552	0.9731	0.7084	0.9317	0.8048
	**RR → S1**	0.9956	0.9747	0.9850	0.8163	0.9630	0.8836
	**RR → S2**	0.9921	0.9674	0.9796	0.7701	0.9341	0.8442
	**RR → Both**	0.9960	0.9731	0.9844	0.8078	0.9663	0.8800
**FL**	**Baseline**	0.9917	0.9680	0.9797	0.7728	0.9308	0.8445
	**RR → S1**	0.9949	0.9747	0.9847	0.8154	0.9573	0.8807
	**RR → S2**	0.9915	0.9748	0.9831	0.8115	0.9289	0.8662
	**RR → Both**	0.9946	0.9781	0.9863	0.8360	0.9550	0.8915

**Table 8:** Stage-2 performance across all configurations.

		N			SVEB			VEB			F		
		Pr	Re	F1	Pr	Re	F1	Pr	Re	F1	Pr	Re	F1
**CE**	**Baseline**	0.9908	0.9871	0.9890	0.8194	0.7899	0.8044	0.9419	0.9433	0.9426	0.5761	0.8805	0.6965
	**RR → S1**	0.9937	0.9946	0.9941	0.9183	0.8671	0.8920	0.9746	0.9641	0.9693	0.7090	0.8428	0.7701
	**RR → S2**	0.9916	0.9953	0.9934	0.9240	0.8079	0.8621	0.9666	0.9555	0.9610	0.7697	0.8616	0.8131
	**RR → Both**	0.9951	0.9937	0.9944	0.8993	0.8977	0.8985	0.9642	0.9663	0.9652	0.7363	0.8428	0.7859
**FL**	**Baseline**	0.9907	0.9930	0.9919	0.8652	0.7953	0.8288	0.9712	0.9440	0.9574	0.6766	0.8553	0.7556
	**RR → S1**	0.9929	0.9945	0.9937	0.9109	0.8438	0.8760	0.9630	0.9541	0.9585	0.7322	0.8428	0.7836
	**RR → S2**	0.9905	0.9960	0.9932	0.9266	0.7935	0.8549	0.9757	0.9519	0.9637	0.7586	0.8302	0.7928
	**RR → Both**	0.9939	0.9941	0.9940	0.9104	0.8761	0.8930	0.9681	0.9598	0.9640	0.7143	0.8491	0.7759

**Table 9:** Wilcoxon signed-rank test results for the contribution of RR-interval features (one-sided, paired across 6 folds). Δ is the mean difference in Stage-2 macro F1 between the RR-augmented configuration and its no-RR baseline. With n=6, the smallest achievable one-sided *p*-value is 0.016. * p<0.05; n.s. = not significant.

Comparison	Δ Stage-2 F1_macro_	*p* (One-Sided)	Significance
CE:No RR vs. CE:RR→S1	+0.029	0.016	*
CE:No RR vs. CE:RR→S2	+0.013	0.078	n.s.
CE:No RR vs. CE:RR→Both	+0.034	0.016	*
FL:No RR vs. FL:RR→S1	+0.016	0.016	*
FL:No RR vs. FL:RR→S2	+0.010	0.219	n.s.
FL:No RR vs. FL:RR→Both	+0.028	0.016	*

**Table 10:** Wilcoxon signed-rank test results comparing Focal Loss against Cross-Entropy at each routing configuration (two-sided, paired across 6 folds). Δ is the mean difference in Stage-2 macro F1 (FL − CE). With n=6, the smallest achievable two-sided *p*-value is 0.031. * p<0.05; n.s. = not significant.

Routing Configuration	Δ Stage-2 F1_macro_ (FL − CE)	*p* (Two-Sided)	Significance
No RR (Baseline)	+0.013	0.031	*
RR→S1	+0.000	0.844	n.s.
RR→S2	+0.009	0.219	n.s.
RR→Both	+0.007	0.438	n.s.

**Table 11:** FL–CE performance gap (ΔF1 = FL_F1_− CE_F1_) across routing configurations, measured using macro-averaged F1. Positive values indicate FL outperforms CE; negative values indicate CE outperforms FL.

Configuration	Stage-1 ΔF1	Stage-2 ΔF1
Baseline (No RR)	+2.31%	+2.53%
RR→S1	−0.16%	−0.34%
RR→S2	+1.28%	−0.62%
RR→Both	+0.67%	−0.43%

**Table 12:** Key criteria for wearable continuous monitoring deployment.

Criterion	Priority
Don’t miss arrhythmias (Abnormal recall) ^a^	Critical
VEB detection (clinically dangerous) ^b^	Critical
SVEB detection (AF predictor) ^c^	High
Minimize alarm fatigue (Abnormal precision) ^d^	High
Overall balance (Stage-2 Overall F1)	High
Computational efficiency (percentage routed) ^e^	Moderate

^a^ [48,49,50]; ^b^ [51,52]; ^c^ [53]; ^d^ [54,55]; ^e^ [56].

**Table 13:** CE:RR→Both performance metrics.

	Stage-1		Stage-2			
	Normal	Abnormal	↓*(If Abnormal)*			
			N	SVEB	VEB	F
**Pr**	0.9960	0.8078	0.9951	0.8993	0.9642	0.7363
**Re**	0.9731	0.9663	0.9937	0.8977	0.9663	0.8428
**F1**	0.9844	0.8800	0.9944	0.8985	0.9652	0.7859

**Table 14:** CE:RR→Both classification performance on PYNQ-Z2 FPGA.

	Stage-1		Stage-2			
	Normal	Abnormal	↓*(If Abnormal)*			
			N	SVEB	VEB	F
**Pr**	0.9961	0.7867	0.9951	0.8715	0.9621	0.6701
**Re**	0.9694	0.9673	0.9916	0.9013	0.9663	0.8302
**F1**	0.9825	0.8677	0.9934	0.8861	0.9642	0.7416

**Table 15:** Two-stage QCSNN resource utilization.

Resource	Used	Available	Utilization (%)
LUT	19,580	53,200	36.80
FF	23,646	106,400	22.22
DSP	220	220	100.00
BRAM	188	280	67.14

*Device: Xilinx Zynq-7020 (PYNQ-Z2)*; *Clock Frequency: 100 MHz*.

**Table 16:** Two-stage QCSNN: power, latency and energy comparison.

Metric	GPU	FPGA	GPU/FPGA
Power Consumption (W)	63.96	2.02	31.66
Time per Inference (ms)	1.463	11.543	0.127
Energy per Inference (mJ)	93.55	23.31	0.36
Throughput (samples/s)	683.72	86.63	7.89

**Table 17:** Deployment validation summary: GPU vs. FPGA.

Metric	GPU	FPGA
Stage-1 Macro F1	93.22%	92.51%
Stage-2 Macro F1	91.10%	89.63%
Latency (ms/beat)	1.46	11.54
Throughput (samples/s)	683.72	86.63
Power (W)	63.96	2.02
Energy/Inference (mJ)	93.55	23.31

**Table 18:** Model architecture and classification performance on MIT-BIH.

Model	Type	Eval.	Params	Size (MB)	Acc. (%)
HARDC [60]	DNN	Intra	223,109 ^a^	∼0.85 ^b^	99.01
CNN-BiLSTM [61]	DNN	Intra	634,621	∼2.42 ^b^	99.21
SNN+CAM [32]	SNN	Intra	435,336	∼1.66 ^b^	98.26
Spike-driven SNN [6]	SNN	Intra	–	0.0126	98.22
All-Spiking SNN [62]	SNN	Intra ^c^	–	–	98.40
PCGQ-CNN [63]	CNN	–	34,273	–	97.98
**QCSNN (Ours)**	**SNN**	**Intra**	**65,539**	**0.064**	**99.02**

^a^ Estimated from architecture. ^b^ Estimated at FP32 (4 bytes/parameter). ^c^ 70/10/20 train/validation/test split.

**Table 19:** Hardware-deployed ECG classification models on MIT-BIH.

Model	Quant.	Platform	Latency	Power	Energy/Inf.
SNN+CAM [32]	No	FPGA	1.37 ms	246 mW	346 μJ
Spike-driven SNN [6]	6-bit	ASIC ^a^	–	0.93 μW	0.75 μJ
All-Spiking SNN [62]	8-bit	iCE40 FPGA	4.32 ms	11.8 mW	50.98 μJ
PCGQ-CNN [63]	INT8/16	PYNQ-Z2	45.1 ms ^b^	–	–
**QCSNN (Ours)**	**INT8**	**PYNQ-Z2**	**11.54 ms** ^c^	**0.33 W** ^d^	**23.31 mJ**

^a^ Validated on FPGA; ASIC results reported in 40 nm CMOS. ^b^ HLS-calculated latency (clock cycles × period); not runtime-measured. ^c^ Runtime-measured on PYNQ-Z2 via Python, including DMA and PS overhead. ^d^ Accelerator power only; total board power is 2.02 W.

## Data Availability

The MIT-BIH dataset is publicly available on the website “PhysioNet”. Code is publicly available.

## Supplementary Materials

The following supporting information can be downloaded at: https://www.mdpi.com/article/10.3390/s26123723/s1. Table S1: Substantive extensions of the present work relative to our previous conference publication; Table S2: Per-class precision, recall, and F1-score for the CE:RR→Both deployment configuration on the intra-patient test set, reported across all six cross-validation folds as mean ± standard deviation.
