# Supplementary material for: A Cascaded Quantized Spiking Neural Network for Real-Time ECG Arrhythmia Detection on Edge Hardware
Source: Sensors (Basel). 2026 Jun 11;26(12):3723. doi: 10.3390/s26123723 (PMC13306364; doi:10.3390/s26123723)
Supplement: Supplementary file 1 [file sensors-26-03723-s001.zip › sensors-4315404-supplementary-english.pdf]

Supplementary Materials: A Cascaded Quantized Spiking Neural Network for Real-Time ECG Arrhythmia Detection on Edge Hardware

Olamilekan Banjo and Behnaz Ghoraani *

This supplementary document provides additional context to the main manuscript through two tables: a structured comparison between this work and our previous conference publication (Table S1), and the per-class per-fold metrics for the deployed CE:RR→Both configuration on the intra-patient test set (Table S2).

Table S1. Substantive extensions of the present work relative to our previous conference publication [1].

**Dimension**	**Previous work [1]**	**Present work**
Training architecture	Binary and four-class stages trained independently as two separate networks.	Jointly trained dual-head architecture sharing a single convolutional backbone, co-adapted via a combined loss in a single optimization process.
Loss–feature interaction study	Single fixed combination of loss function and input features; no ablation.	Eight-configuration factorial study (two loss functions × four RR-routing strategies) surfacing the interaction effect that is among the central findings of this work.
FPGA deployment and validation	No hardware deployment.	PYNQ-Z2 FPGA deployment with hardware-resident cascade early termination; comprehensive GPU–FPGA comparisons across classification fidelity, latency, throughput, power, and resource utilization.
RR-interval features	Morphological waveform features only.	Additionally incorporates four RR-interval features (RR_{prev}, RR_{next}, RR_{ratio}, RR_{diff}) and demonstrates their interaction with loss function choice.

Table S2. Per-class precision, recall, and F1-score for the CE:RR→Both deployment configuration on the intra-patient test set, reported across all six cross-validation folds as mean ± standard deviation.

Class	Precision	Recall	F1-score
Stage-1 (binary: Normal vs. Abnormal)			
Normal	0.995 ± 0.000	0.972 ± 0.003	0.984 ± 0.002
Abnormal	0.801 ± 0.017	0.961 ± 0.002	0.874 ± 0.010
Stage-2 (four-class: N, SVEB, VEB, F)			
N (Normal)	0.994 ± 0.001	0.992 ± 0.003	0.993 ± 0.001
SVEB	0.854 ± 0.041	0.881 ± 0.014	0.866 ± 0.018
VEB	0.965 ± 0.008	0.959 ± 0.004	0.962 ± 0.005
F (Fusion)	0.732 ± 0.069	0.851 ± 0.022	0.785 ± 0.039

1. Banjo, O.; Ghoraani, B. QCSNN: A Memory-Efficient Spiking Neural Network for On-Device ECG-Based Arrhythmia Detection. In Proceedings of the IEEE International Conference on Omni-layer Intelligent Systems (COINS), Madison, WI, USA, 2025; pp. 1–6.
