# Supplementary figures and images for: A Cascaded Quantized Spiking Neural Network for Real-Time ECG Arrhythmia Detection on Edge Hardware
Source: Sensors (Basel). 2026 Jun 11;26(12):3723. doi: 10.3390/s26123723 (PMC13306364; doi:10.3390/s26123723)

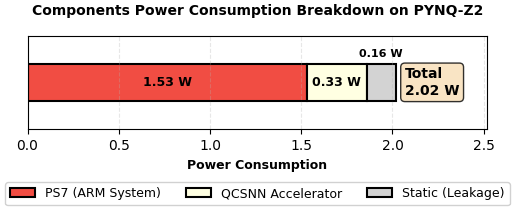

Supplement: Supplementary file 1 [file sensors-26-03723-s001.zip › Definitions/fig_14_qcsnn24_fused_power.png]

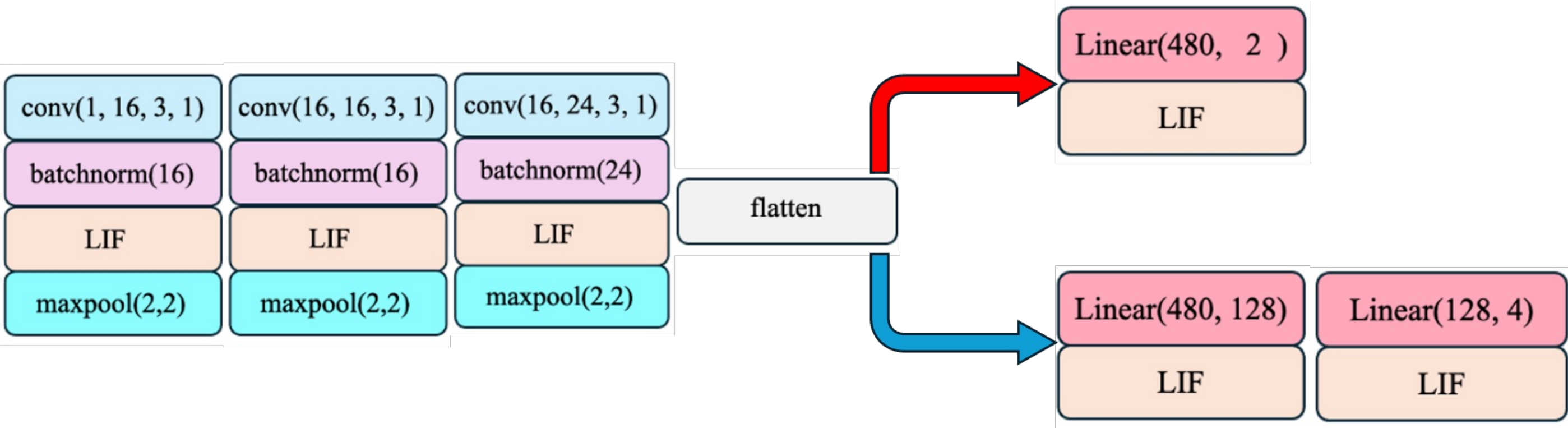

Supplement: Supplementary file 1 [file sensors-26-03723-s001.zip › Definitions/fig_1_qcsnn24_fused_architecture.pdf]

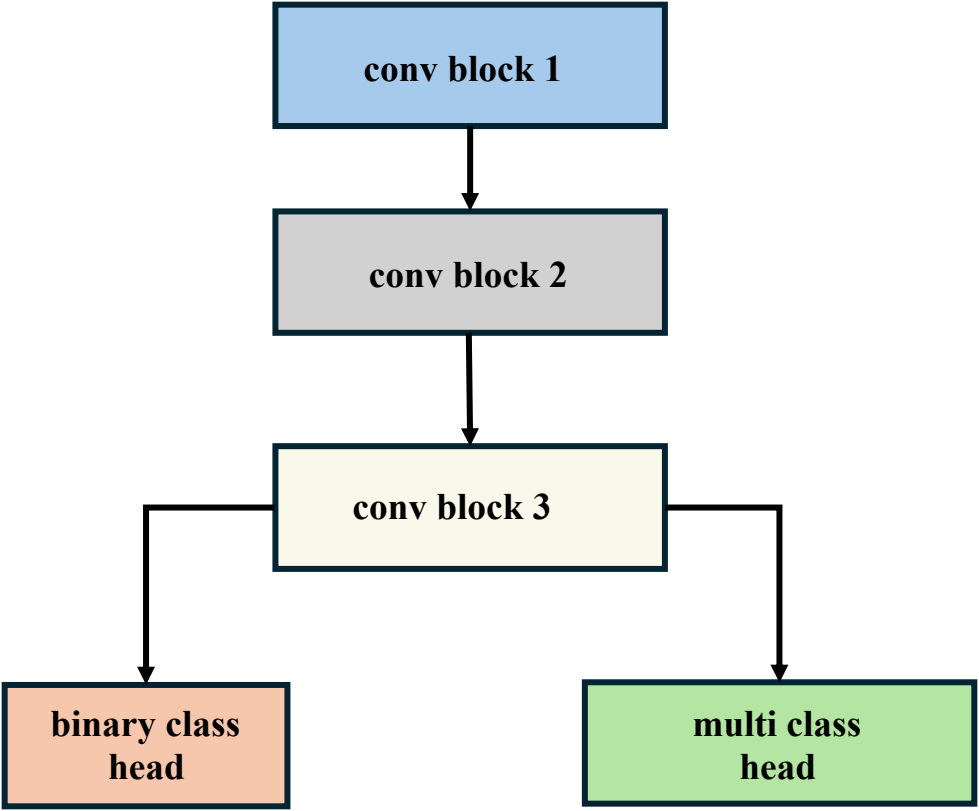

Supplement: Supplementary file 1 [file sensors-26-03723-s001.zip › Definitions/fig_2_fused_stages_training.pdf]

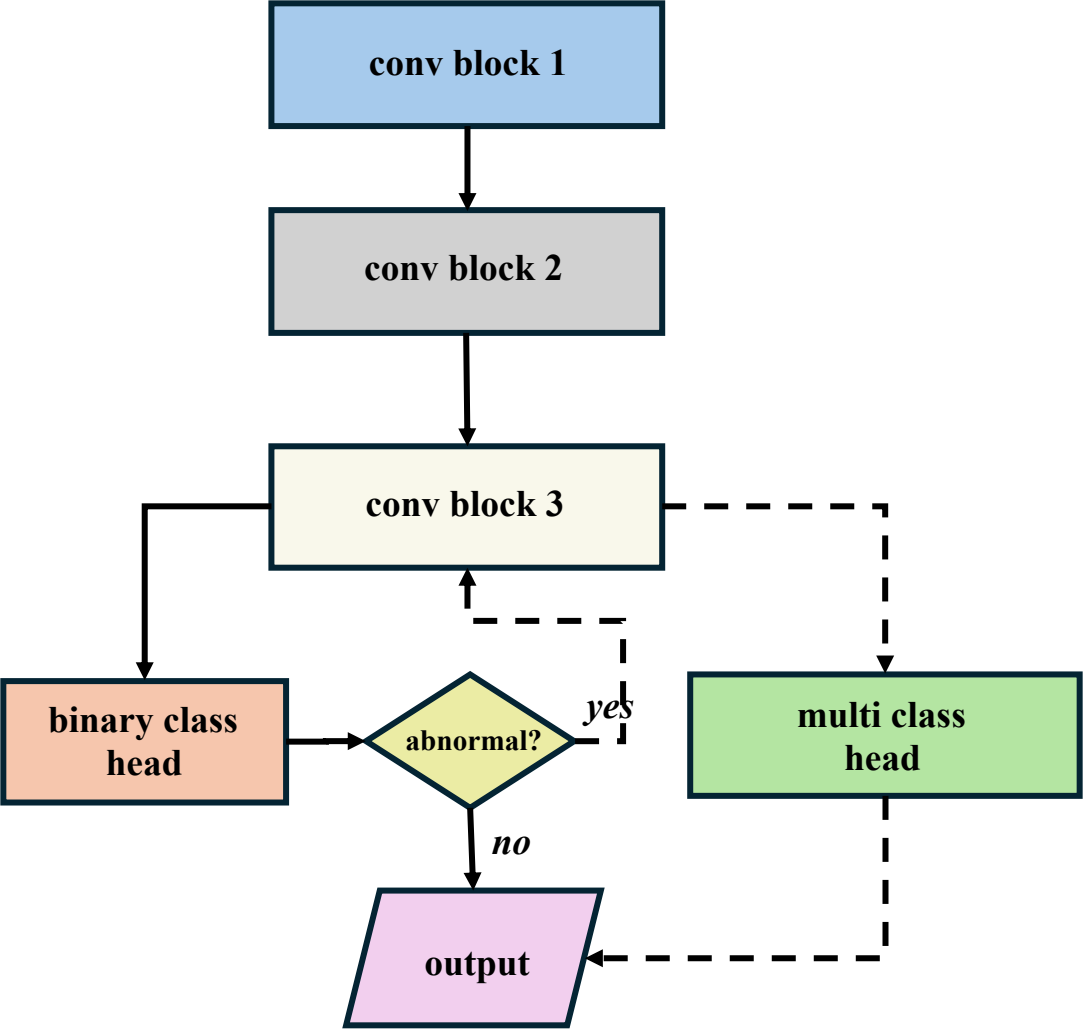

Supplement: Supplementary file 1 [file sensors-26-03723-s001.zip › Definitions/fig_3_fused_stages_inference.pdf]

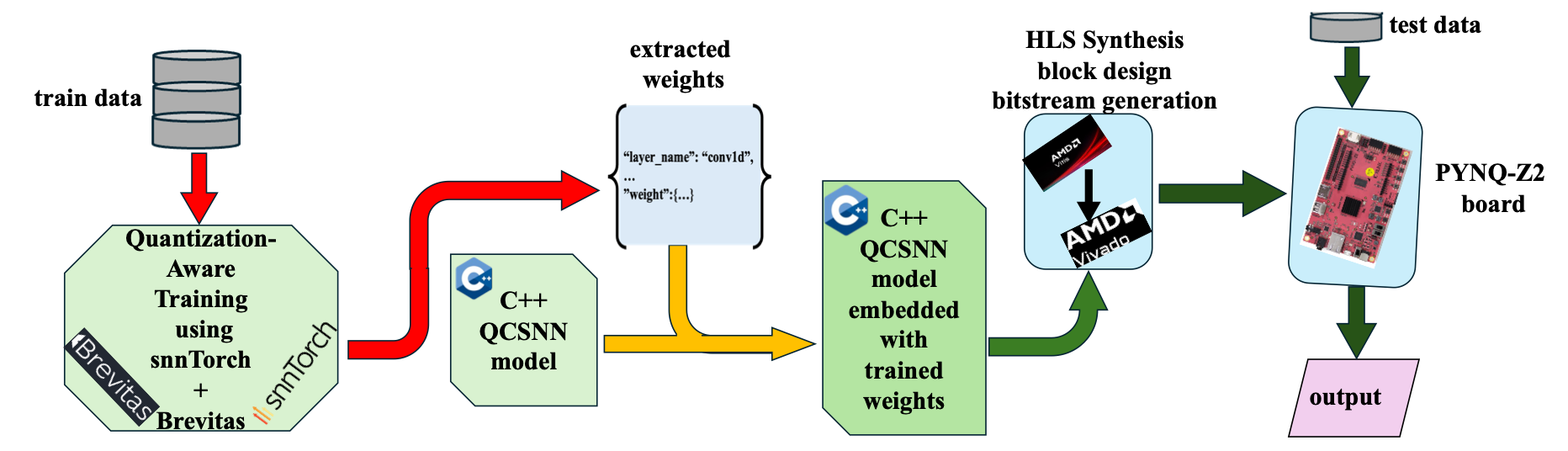

Supplement: Supplementary file 1 [file sensors-26-03723-s001.zip › Definitions/fig_4_soft_hard_v3.png]

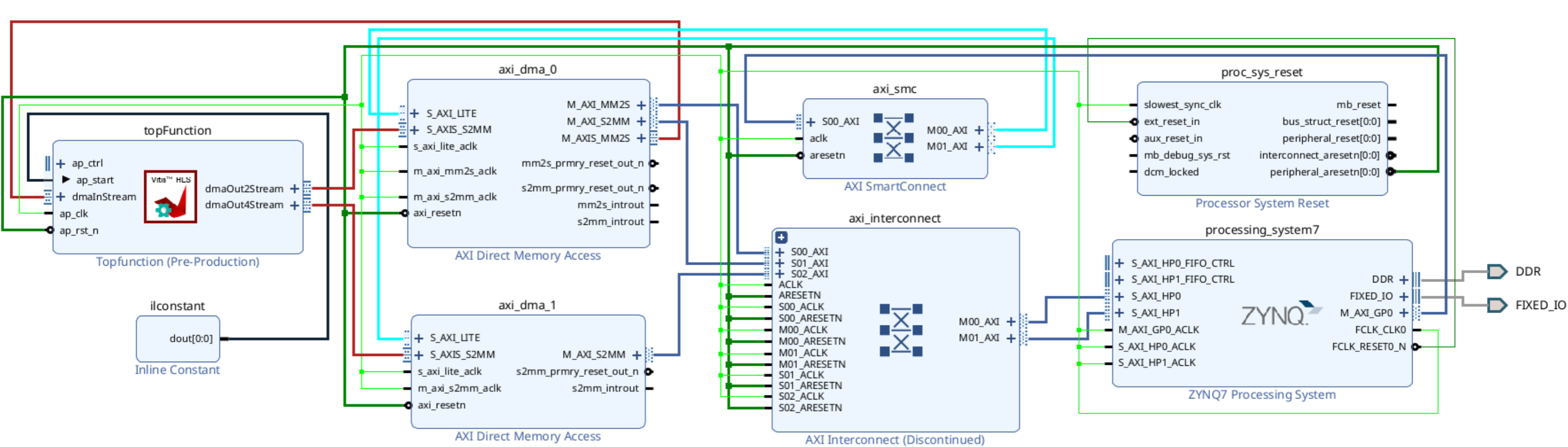

Supplement: Supplementary file 1 [file sensors-26-03723-s001.zip › Definitions/fig_5_qcsnn24_fused_bd.pdf]

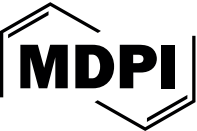

Supplement: Supplementary file 1 [file sensors-26-03723-s001.zip › Definitions/logo-mdpi-eps-converted-to.pdf]

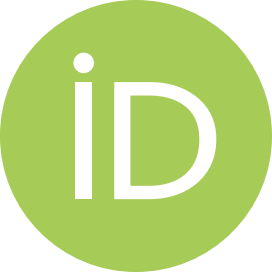

Supplement: Supplementary file 1 [file sensors-26-03723-s001.zip › Definitions/logo-orcid.pdf]

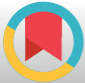

Check for updates

Supplement: Supplementary file 1 [file sensors-26-03723-s001.zip › Definitions/logo-updates-eps-converted-to.pdf]

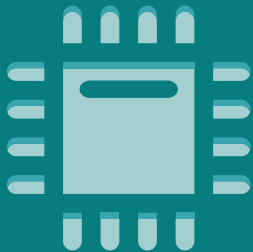

*sensors*

Supplement: Supplementary file 1 [file sensors-26-03723-s001.zip › Definitions/sensors-logo-eps-converted-to.pdf]

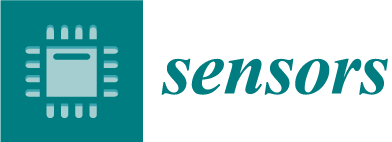

Supplement: Supplementary file 1 [file sensors-26-03723-s001.zip › Definitions/sensors-logo.png]
